# Exponential Strong Converse for Source Coding with Side Information at the Decoder †

**DOI:** 10.3390/e20050352

**Published:** 2018-05-08

**Authors:** Yasutada Oohama

**Affiliations:** Department of Communication Engineering and Informatics, University of Electro-Communications, Tokyo 182-8585, Japan; oohama@uec.ac.jp; Tel.: +81-42-443-5358

**Keywords:** source coding with side information at the decoder, the rate distortion region, exponent function outside the rate distortion region, strong converse theorem

## Abstract

We consider the rate distortion problem with side information at the decoder posed and investigated by Wyner and Ziv. Using side information and encoded original data, the decoder must reconstruct the original data with an arbitrary prescribed distortion level. The rate distortion region indicating the trade-off between a data compression rate *R* and a prescribed distortion level Δ was determined by Wyner and Ziv. In this paper, we study the error probability of decoding for pairs of (R,Δ) outside the rate distortion region. We evaluate the probability of decoding such that the estimation of source outputs by the decoder has a distortion not exceeding a prescribed distortion level Δ. We prove that, when (R,Δ) is outside the rate distortion region, this probability goes to zero exponentially and derive an explicit lower bound of this exponent function. On the Wyner–Ziv source coding problem the strong converse coding theorem has not been established yet. We prove this as a simple corollary of our result.

## 1. Introduction

For single or multi terminal source coding systems, the converse coding theorems state that at any data compression rates below the fundamental theoretical limit of the system the error probability of decoding *cannot go to zero* when the block length *n* of the codes tends to infinity. On the other hand, the strong converse theorems state that, at any transmission rates exceeding the fundamental theoretical limit, the error probability of decoding *must go to one* when *n* tends to infinity. The former converse theorems are sometimes called the weak converse theorems to distinguish them with the strong converse theorems.

**Figure 1:**
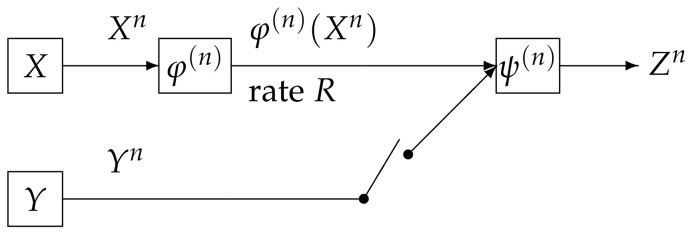
Source encoding with a fidelity criterion with or without side inforamion at the decoder.

In this paper, we study the strong converse theorem for the rate distortion problem with side information at the decoder posed and investigated by Wyner and Ziv [1]. We call the above source coding system the Wyner and Ziv source coding system (the WZ system). The WZ system is shown in Figure 1. In this figure, the WZ system corresponds to the case where the switch is close. In Figure 1, the sequence $\left(X^{n},Y^{n}\right)$ represents independent copies of a pair of dependent random variables (X,Y) which take values in the finite sets X and Y, respectively. We assume that (X,Y) has a probability distribution denoted by $p_{XY}$. The encoder $\varphi^{\left(n\right)}$ outputs a binary sequence which appears at a rate *R* bits per input symbol. The decoder function $\psi^{\left(n\right)}$ observes $\varphi^{\left(n\right)}\left(X^{n}\right)$ and $Y^{n}$ to output a sequence $Z^{n}$. The *t*-th component $Z_{t}$ of $Z^{n}$ for t=1,2,⋯,n take values in the finite reproduction alphabet Z. Let d:X×Z→[0,∞) be an arbitrary distortion measure on X×Z. The distortion between $x^{n}\in X^{n}$ and $z^{n}\in Z^{n}$ is defined by

$$d\left(x^{n},z^{n}\right):=\sum_{t=1}^{n}d\left(x_{t},z_{t}\right).$$

In general, we have two criteria on $d(X^{n},Z^{n})$. One is the excess-distortion probability of decoding defined by

(1) $$P_{e}^{\left(n\right)}\left(\varphi^{\left(n\right)},\psi^{\left(n\right)};\Delta \right):=\mathrm{Pr}\left\{\frac{1}{n}d\left(X^{n},Z^{n}\right)\geq \Delta \right\}.$$

The other is the average distortion defined by

$$\begin{array}{cc} \Delta_{n}:= & E\left[\frac{1}{n}d\left(X_{,}^{n}Z^{n}\right)\right]:=\sum_{\left(x^{n},z^{n}\right)\in X^{n}\times Z^{n}}\left[\frac{1}{n}\sum_{k=1}^{n}d\left(x_{k},z_{k}\right)\right]\mathrm{Pr}\left\{X^{n}=x^{n},Z^{n}=z^{n}\right\}\\ = & \frac{1}{n}\sum_{k=1}^{n}\sum_{(x_{k},z_{k})\in X\times Z}d\left(x_{k},z_{k}\right)\mathrm{Pr}\left\{X_{k}=x_{k},Z_{k}=z_{k}\right\}. \end{array}$$

A pair (R,Δ) is ε-*achievable* for $p_{XY}$ if there exist a sequence of pairs $\{(\varphi^{\left(n\right)},$ $\psi^{\left(n\right)}{)\}}_{n\geq 1}$ such that for any δ>0 and any *n* with $n\geq n_{0}=n_{0}\left(\varepsilon ,\delta \right)$

$$\begin{array}{c} \frac{1}{n}\log∥\varphi^{\left(n\right)}∥\leq R+\delta ,\;P_{e}^{\left(n\right)}\left(\varphi^{\left(n\right)},\psi^{\left(n\right)};\Delta \right)\leq \varepsilon , \end{array}$$

where $∥\varphi^{\left(n\right)}∥$ stands for the range of cardinality of $\varphi^{\left(n\right)}$. The rate distortion region $R_{\mathrm{WZ}}\left(\varepsilon |p_{XY}\right)$ is defined by

$$\begin{array}{ccc} R_{\mathrm{WZ}}\left(\varepsilon |p_{XY}\right) & = & \left\{\;\left(R,\Delta \right):\left(R,\Delta \right)\;\mathrm{is}\;\varepsilon \text{-}\mathrm{achievable}\;\mathrm{for}\;p_{XY}\;\right\}. \end{array}$$

Furthermore, set

$$R_{\mathrm{WZ}}\left(p_{XY}\right):=\underset{\varepsilon >0}{⋂}R_{\mathrm{WZ}}\left(\varepsilon |p_{XY}\right).$$

On the other hand, we can define a rate distortion region based on the average distortion criterion, a formal definition of which is the following. A pair (R,Δ) is *achievable* for $p_{XY}$ if there exist a sequence of pairs $\{(\varphi^{\left(n\right)},$$\psi^{\left(n\right)}{)\}}_{n\geq 1}$ such that for any δ>0 and any *n* with $n\geq n_{0}$$=n_{0}\left(\delta \right)$,

$$\begin{array}{c} \frac{1}{n}\log∥\varphi^{\left(n\right)}∥\leq R+\delta ,\;\Delta^{\left(n\right)}\leq \Delta +\delta . \end{array}$$

The rate distortion region ${\tilde{R}}_{\mathrm{WZ}}\left(p_{XY}\right)$ is defined by

$$\begin{array}{ccc} {\tilde{R}}_{\mathrm{WZ}}\left(p_{XY}\right) & := & \left\{\;\left(R,\Delta \right):\left(R,\Delta \right)\;\mathrm{is}\;\mathrm{achievable}\;\mathrm{for}\;p_{XY}\;\right\}. \end{array}$$

If the switch is open, then the side information is not available to the decoder. In this case the communication system corresponds to the source coding for the discrete memoryless source (DMS) specified with $p_{X}$. We define the rate distortion region ${\tilde{R}}_{\mathrm{DMS}}\left(p_{X}\right)$ in a similar manner to the definition of ${\tilde{R}}_{\mathrm{WZ}}\left(p_{XY}\right)$. We further define the region $R_{\mathrm{DMS}}\left(\varepsilon |p_{X}\right),\varepsilon \in \left(0,1\right)$ and $R_{\mathrm{DMS}}\left(p_{X}\right)$, respectively in a similar manner to the definition of $R_{\mathrm{WZ}}\left(\varepsilon |p_{XY}\right)$ and $R_{\mathrm{WZ}}\left(p_{XY}\right)$.

Previous works on the characterizations of ${\tilde{R}}_{\mathrm{DMS}}\left(p_{X}\right)$, $R_{\mathrm{DMS}}\left(\varepsilon |p_{X}\right),\varepsilon \in \left(0,1\right)$, and $R_{\mathrm{DMS}}\left(p_{X}\right)$ are shown in Table 1. Shannon [2] determined ${\tilde{R}}_{\mathrm{DMS}}\left(p_{X}\right)$. Subsequently, Wolfowiz [3] proved that ${\tilde{R}}_{\mathrm{DMS}}\left(p_{X}\right)=R_{\mathrm{DMS}}\left(p_{X}\right).$ Furthermore, he proved the strong converse theorem. That is, if (R,Δ)∉ ${\tilde{R}}_{\mathrm{DMS}}\left(p_{X}\right)$, then for any sequence ${\left\{\left(\varphi^{\left(n\right)},\psi^{\left(n\right)}\right)\right\}}_{n=1}^{\infty }$ of encoder and decoder functions satisfying the condition

(2) $$\underset{n\to \infty }{lim sup}\frac{1}{n}\log||\varphi^{\left(n\right)}\left|\right|\leq R,$$

we have

(3) $$\lim_{n\to \infty }P_{e}^{\left(n\right)}\left(\varphi^{\left(n\right)},\psi^{\left(n\right)};\Delta \right)=\lim_{n\to \infty }\mathrm{Pr}\left\{\frac{1}{n_{k}}d\left(X^{n},Z^{n}\right)\geq \Delta \right\}=1.$$

The above strong converse theorem implies that, for any ε∈(0,1),

$${\tilde{R}}_{\mathrm{DMS}}\left(p_{X}\right)=R_{\mathrm{DMS}}\left(p_{X}\right)=R_{\mathrm{DMS}}\left(\varepsilon |p_{X}\right).$$

Csiszár and Körner proved that in Equation (3), the probability $P_{e}^{\left(n\right)}\left(\varphi^{\left(n\right)},\psi^{\left(n\right)};\Delta \right)$ converges to one exponentially and determined the optimal exponent as a function of (R,Δ).

The previous works on the coding theorems for the WZ system are summarized in Table 1. The rate distortion region ${\tilde{R}}_{\mathrm{WZ}}\left(p_{XY}\right)$ was determined by Wyner and Ziv [1]. Csiszár and Körner [4] proved that ${\tilde{R}}_{\mathrm{WZ}}\left(p_{XY}\right)$ $=R_{\mathrm{WZ}}\left(p_{XY}\right)$. On the other hand, we have had no result on the strong converse theorem for the WZ system.

Main results of this paper are summarized in Table 1. For the WZ system, we prove that if (R,Δ) is out side the rate distortion region $R_{\mathrm{WZ}}\left(p_{XY}\right)$, then we have that for any sequence ${\left\{\left(\varphi^{\left(n\right)},\psi^{\left(n\right)}\right)\right\}}_{n=1}^{\infty }$ of encoder and decoder functions satisfying the conditionin Equation (2), the quantity $P_{e}^{\left(n\right)}\left(\varphi^{\left(n\right)},\psi^{\left(n\right)};\Delta \right)$ goes to zero exponentially and derive an explicit lower bound of this exponent function. This result corresponds to Theorem 3 in Table 1. As a corollary from this theorem, we obtain the strong converse result, which is stated in Corollary 2 in Table 1. This results states that we have an outer bound with $O(1\sqrt{n})$ gap from the rate distortion region $R_{\mathrm{WZ}}\left(p_{XY}\right)$.

To derive our result, we use a new method called the recursive method. This method is a general powerful tool to prove strong converse theorems for several coding problems in information theory. In fact, the recursive method plays important roles in deriving exponential strong converse exponent for communication systems treated in [5,6,7,8].

## 2. Source Coding with Side Information at the Decoder

In the following argument, the operations $E_{p}\left[\cdot \right]$ and ${\mathrm{Var}}_{p}\left[\cdot \right]$, respectively, stand for the expectation and the variance with respect to a probability distribution *p*. When the value of *p* is obvious from the context, we omit the suffix *p* in those operations to simply write E[·] and Var[·]. Let X and Y be finite sets and ${\left\{(X_{t},Y_{t})\right\}}_{t=1}^{\infty }$ be a stationary discrete memoryless source. For each t=1,2,⋯, the random pair $\left(X_{t},Y_{t}\right)$ takes values in X×Y, and has a probability distribution

$$p_{XY}={\left\{p_{XY}\left(x,y\right)\right\}}_{(x,y)\in X\times Y}.$$

We write *n* independent copies of ${\left\{X_{t}\right\}}_{t=1}^{\infty }$ and ${\left\{Y_{t}\right\}}_{t=1}^{\infty }$, respectively, as

$$X^{n}=X_{1},X_{2},\cdots ,X_{n}\;\mathrm{and}\;Y^{n}=Y_{1},Y_{2},\cdots ,Y_{n}.$$

**Figure 2:**
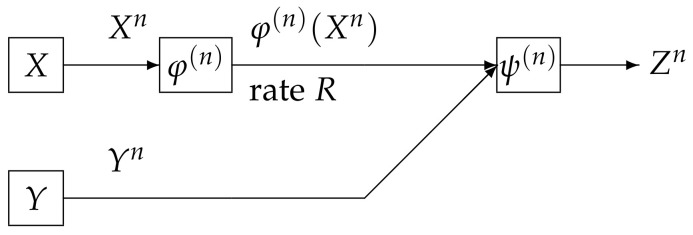
Wyner–Ziv source coding system.

We consider a communication system depicted in Figure 2. Data sequences $X^{n}$ is separately encoded to $\varphi^{\left(n\right)}\left(X^{n}\right)$ and is sent to the information processing center. At the centerm the decoder function $\psi^{\left(n\right)}$ observes $\varphi^{\left(n\right)}\left(X^{n}\right)$ and $Y^{n}$ to output the estimation $Z^{n}$ of $X^{n}$. The encoder function $\varphi^{\left(n\right)}$ is defined by

(4) $$\begin{array}{c} \varphi^{\left(n\right)}:X^{n}\to M_{n}=\left\{\;1,2,\cdots ,M_{n}\;\right\}. \end{array}$$

Let Z be a reproduction alphabet. The decoder function $\psi^{\left(n\right)}$ is defined by

(5) $$\psi^{\left(n\right)}:M_{n}\times Y^{n}\;\to \;Z^{n}.$$

Let d:X×Z→[0,∞) be an arbitrary distortion measure on X×Z. The distortion between $x^{n}\in X^{n}$ and $z^{n}\in Z^{n}$ is defined by

$$d\left(x^{n},z^{n}\right):=\sum_{t=1}^{n}d\left(x_{t},z_{t}\right).$$

The excess-distortion probability of decoding is

(6) $$P_{e}^{\left(n\right)}\left(\varphi^{\left(n\right)},\psi^{\left(n\right)};\Delta \right)=\mathrm{Pr}\left\{\frac{1}{n}d\left(X^{n},Z^{n}\right)\geq \Delta \right\},$$

where $Z^{n}=\psi^{\left(n\right)}\left(\varphi^{\left(n\right)}\left(X^{n}\right),Y^{n}\right)$. The average distortion $\Delta^{\left(n\right)}$ between $X^{n}$ and $Z^{n}$ is defined by

$$\Delta^{\left(n\right)}:=\frac{1}{n}E\left[d(X^{n},Z^{n})\right]:=\frac{1}{n}\sum_{t=1}^{n}Ed\left(X_{t},Z_{t}\right).$$

In the previous section, we gave the formal definitions of $R_{\mathrm{WZ}}\left(\varepsilon \right|$$p_{XY})$, ε∈(0,1), $R_{\mathrm{WZ}}\left(p_{XY}\right)$, and ${\tilde{R}}_{\mathrm{WZ}}\left(p_{XY}\right)$. We can show that the above three rate distortion regions satisfy the following property.

**Property** **1.**

*(a)*  *The regions $R_{\mathrm{WZ}}\left(\varepsilon |p_{XY}\right)$, ε∈(0,1), $R_{\mathrm{WZ}}\left(p_{XY}\right)$, and ${\tilde{R}}_{\mathrm{WZ}}\left(p_{XY}\right)$ are closed convex sets of $R_{+}^{2}$, where*
  $$\begin{array}{ccc} R_{+}^{2} & := & \{(R,\Delta ):R\geq 0,\Delta \geq 0\}. \end{array}$$
*(b)*  *$R_{\mathrm{WZ}}\left(\varepsilon |p_{XY}\right)$ has another form using (n,ε)-rate distortion region, the definition of which is as follows. We set*
  $$\begin{array}{ccc} R_{\mathrm{WZ}}\left(n,\varepsilon |p_{XY}\right) & = & \{\left(R,\Delta \right):There\;exists\left(\varphi^{\left(n\right)},\psi^{\left(n\right)}\right)such\;that\\ & & \frac{1}{n}\log||\varphi^{\left(n\right)}\left|\right|\leq R,\;P_{e}^{\left(n\right)}\left(\varphi^{\left(n\right)},\psi^{\left(n\right)};\Delta \right)\leq \varepsilon \}, \end{array}$$
  *which is called the (n,ε)-rate distortion region. Using $R_{\mathrm{WZ}}\left(n,\varepsilon |p_{XY}\right)$, $R_{\mathrm{WZ}}\left(\varepsilon |p_{XY}\right)$ can be expressed as*
  $$\begin{array}{ccc} R_{\mathrm{WZ}}\left(\varepsilon |p_{XY}\right) & = & \mathrm{cl}\left(\underset{m\geq 1}{⋃}\underset{n\geq m}{⋂}R_{\mathrm{WZ}}\left(n,\varepsilon |p_{XY}\right)\right), \end{array}$$
  *where cl(·) stands for the closure operation.*

Proof of this property is given in Appendix A.

It is well known that ${\tilde{R}}_{\mathrm{WZ}}\left(p_{XY}\right)$ was determined by Wyner and Ziv [1]. To describe their result we introduce auxiliary random variables *U* and *Z*, respectively, taking values in finite sets U and Z. We assume that the joint distribution of (U,X,Y,Z) is

$$\begin{array}{c} p_{UXYZ}\left(u,x,y,z\right)=p_{U}\left(u\right)p_{X|U}\left(x|u\right)p_{Y|X}\left(y|x\right)p_{Z|UY}\left(z|u,y\right). \end{array}$$

The above condition is equivalent to

U↔X↔Y,X↔(U,Y)↔Z.

Define the set of probability distribution $p=p_{UXYZ}$ by

$$\begin{array}{ccc} P(p_{XY}) & := & \{p=p_{UXYZ}:|U|\leq |X|+1,U↔X↔Y,X↔(U,Y)↔Z\},\\ P^{*}\left(p_{XY}\right) & := & \{p=p_{UXYZ}:|U|\leq |X|+1,U↔X↔Y,Z=\phi (U,Y)\mathrm{for}\;\mathrm{some}\phi :U\times Y\to Z\}. \end{array}$$

By definitions, it is obvious that $P^{*}\left(p_{XY}\right)\subseteq P\left(p_{XY}\right)$. Set

$$\begin{array}{ccc} R(p) & := & \begin{array}{c} \left\{ \left(R,\Delta \right):R,\Delta \geq 0\;,\begin{array}{ccc} R & \geq & I_{p}\left(X;U|Y\right),\Delta \geq E_{p}d\left(X,Z\right)\}, \end{array}\right. \end{array}\\ R(p_{XY}) & := & \underset{p\in P(p_{XY})}{⋃}R\left(p\right),R^{*}\left(p_{XY}\right):=\underset{p\in P^{*}\left(p_{XY}\right)}{⋃}R\left(p\right). \end{array}$$

We can show that the above functions and sets satisfy the following property:

**Property** **2.**

*(a)*  *The region $R(p_{XY})$ is a closed convex set of $R_{+}^{2}$.*
*(b)*  *For any $p_{XY}$, we have*
  $$R\left(p_{XY}\right)=R^{*}\left(p_{XY}\right).$$

Proof of Property 2 is given in Appendix C. In Property 2 Part (b), $R(p_{XY})$ is regarded as another expression of $R^{*}\left(p_{XY}\right)$. This expression is useful for deriving our main result. The rate region $R_{\mathrm{WZ}}\left(p_{XY}\right)$ was determined by Wyner and Ziv [1]. Their result is the following:

**Theorem** **1**  (Wyner and Ziv [1])**.**

$$\begin{array}{c} {\tilde{R}}_{\mathrm{WZ}}\left(p_{XY}\right)=R^{*}\left(p_{XY}\right)=R\left(p_{XY}\right). \end{array}$$

On $R_{\mathrm{WZ}}\left(p_{XY}\right)$, Csiszár and Körner [4] obtained the following result.

**Theorem** **2**  (Csiszár and Körner [4])**.**

$$\begin{array}{c} R_{\mathrm{WZ}}\left(p_{XY}\right)={\tilde{R}}_{\mathrm{WZ}}\left(p_{XY}\right)=R^{*}\left(p_{XY}\right)=R\left(p_{XY}\right). \end{array}$$

We are interested in an asymptotic behavior of the error probability of decoding to tend to one as n→∞ for $\left(R,\Delta \right)\notin R_{\mathrm{WZ}}\left(p_{XY}\right)$. To examine the rate of convergence, we define the following quantity. Set

Pc(n)(φ(n),ψ(n);Δ):=1−Pe(n)(φ(n),ψ(n);Δ),G(n)(R,Δ|pXY):=min(φ(n),ψ(n)):(1/n)log∥φ(n)∥≤R−1nlogPc(n)(φ(n),ψ(n);Δ).

By time sharing, we have that

(7) $$\begin{array}{ccc} G^{\left(n+m\right)}\left(\left.\frac{nR+mR^{\prime }}{n+m},\frac{n\Delta +m\Delta^{\prime }}{n+m}\right|p_{XY}\right) & \leq & \frac{nG^{\left(n\right)}\left(R,\Delta |p_{XY}\right)+mG^{\left(m\right)}\left(R^{\prime },\Delta^{\prime }|p_{XY}\right)}{n+m}. \end{array}$$

Choosing $R=R^{\prime }$ and $\Delta =\Delta^{\prime }$ in Equation (7), we obtain the following subadditivity property on $\{G^{\left(n\right)}\left(R,\Delta |p_{XY}\right)$${\}}_{n\geq 1}$:

$$\begin{array}{ccc} G^{\left(n+m\right)}\left(R,\Delta |p_{XY}\right) & \leq & \frac{nG^{\left(n\right)}\left(R,\Delta |p_{XY}\right)+mG^{\left(m\right)}\left(R,\Delta |p_{XY}\right)}{n+m}, \end{array}$$

which together with Fekete’s lemma yields that $G^{\left(n\right)}(R,$$\Delta |p_{XY})$ exists and satisfies the following:

$$\begin{array}{c} \lim_{n\to \infty }G^{\left(n\right)}\left(R,\Delta |p_{XY}\right)=\underset{n\geq 1}{\inf}G^{\left(n\right)}\left(R,\Delta |p_{XY}\right). \end{array}$$

Set

$$\begin{array}{c} G\left(R,\Delta |p_{XY}\right):=\lim_{n\to \infty }G^{\left(n\right)}\left(R,\Delta |p_{XY}\right),\\ G\left(p_{XY}\right):=\left\{\left(R,\Delta ,G\right):G\geq G\left(R,\Delta |p_{XY}\right)\right\}. \end{array}$$

The exponent function $G(R,\Delta |p_{XY})$ is a convex function of (R,Δ). In fact, from Equation (7), we have that for any α∈[0,1]

$$\begin{array}{ccc} G(\alpha R+\bar{\alpha }R^{\prime },\alpha \Delta +\bar{\alpha }\Delta^{\prime }|p_{XY}) & \leq & \alpha G\left(R,\Delta |p_{XY}\right)+\bar{\alpha }G\left(R^{\prime },\Delta^{\prime }|p_{XY}\right), \end{array}$$

where $\bar{\alpha }=1-\alpha$. The region $G(p_{XY})$ is also a closed convex set. Our main aim is to find an explicit characterization of $G(p_{XY})$. In this paper, we derive an explicit outer bound of G $\left(p_{XY}\right)$ whose section by the plane G=0 coincides with $R_{\mathrm{WZ}}\left(p_{XY}\right)$.

## 3. Main Results

In this section, we state our main results. We first explain that the rate distortion region $R(p_{XY})$ can be expressed with two families of supporting hyperplanes. To describe this result, we define two sets of probability distributions on U×X×Y×Z by

$$\begin{array}{ccc} P_{\mathrm{sh}}\left(p_{XY}\right) & := & \{p_{UXYZ}:|U|\leq |X|,U↔X↔Y,X↔\left(U,Y\right)↔Z\}.\\ Q & := & \{q=q_{UXYZ}:|U|\leq |X|\}. \end{array}$$

Let $\bar{\mu }=1-\mu$. We set

$$\begin{array}{c} R^{\left(\mu \right)}\left(p_{XY}\right):=\min_{p\in P_{\mathrm{sh}}\left(p_{XY}\right)}\left\{\bar{\mu }I_{p}\left(X;U|Y\right)+\mu E_{p}d\left(X;Z\right)\right\},\\ R_{\mathrm{sh}}\left(p_{XY}\right):=\underset{\mu \in [0,1]}{⋂}\left\{\left(R,\Delta \right):\bar{\mu }R+\mu \Delta \geq R^{\left(\mu \right)}\left(p_{XY}\right)\right\}. \end{array}$$

Then, we have the following property:

**Property** **3.** *For any $p_{XY}$, we have*

(8) $$R_{\mathrm{sh}}\left(p_{XY}\right)=R\left(p_{XY}\right).$$

Proof of Property 3 is given in Appendix D. For μ∈[0,1] and λ,α≥0, define

ωq||p(μ,λ)(x,y,z|u):=logqX(x)qY|XU(y|x,u)qZ|UYX(z|u,y,x)pX(x)pY|X(y|x)qZ|UY(z|u,y)+λμ¯logqX|YU(x|y,u)pX|Y(x|y)+μd(x,z),Ω(μ,λ,α)(q|pXY):=−logEqexp−αωq||p(μ,λ)(X,Y,Z|U),Ω(μ,λ,α)(pXY):=minq∈QΩ(μ,λ,α)(q|pXY),F(μ,λ,α)(μ¯R+μΔ|pXY):=Ω(μ,λ,α)(pXY)−λα(μ¯R+μΔ)1+(4+λμ¯)α.

Furthermore, set

$$\begin{array}{c} F\left(R,\Delta |p_{XY}\right):=\underset{\mu \in [0,1],\lambda ,\alpha \geq 0}{\sup}F^{\left(\mu ,\lambda ,\alpha \right)}\left(\bar{\mu }R+\mu \Delta |p_{XY}\right),\\ \bar{G}\left(p_{XY}\right):=\left\{\left(R,\Delta ,G\right):G\geq F\left(R,\Delta |p_{XY}\right)\right\}. \end{array}$$

We next define a functions serving as a lower bound of $F(R,\Delta |p_{XY})$. For each $p=p_{UXYZ}\in P_{\mathrm{sh}}\left(p_{XY}\right)$, define

$$\begin{array}{c} {\tilde{\omega }}_{p}^{\left(\mu \right)}\left(x,y,z|u\right):=\left[\bar{\mu }\log\frac{p_{X|YU}\left(x|y,u\right)}{p_{X|Y}\left(x|y\right)}+\mu d\left(x,z\right)\right],\\ {\tilde{\Omega }}^{\left(\mu ,\lambda \right)}\left(p\right):=-\log E_{p}\left[\exp\left\{-\lambda \omega_{p}^{\left(\mu \right)}\left(X,Y,Z|U\right)\right\}\right]. \end{array}$$

Furthermore, set

Ω˜(μ,λ)(pXY):=minp∈Psh(pXY)Ω˜(μ,λ)(p),F˜(μ,λ)(μR+μ¯Δ|pXY):=Ω˜(μ,λ)(pXY)−λ(μ¯R+μΔ)5+λ(1+μ¯),

$$\begin{array}{c} \tilde{F}\left(R,\Delta |p_{XY}\right):=\underset{\lambda \geq 0,\mu \in [0,1]}{\sup}{\tilde{F}}^{\left(\mu ,\lambda \right)}\left(\bar{\mu }R+\mu \Delta |p_{XY}\right). \end{array}$$

We can show that the above functions satisfies the following properties:

**Property** **4.**

*(a)*  *The cardinality bound |U|≤|X| appearing in Q is sufficient to describe the quantity $\Omega^{\left(\mu ,\lambda ,\alpha \right)}\left(p_{XY}\right)$. Furthermore, the cardinality bound |U|≤|X| in $P_{\mathrm{sh}}\left(p_{XY}\right)$ is sufficient to describe the quantity ${\tilde{\Omega }}^{\left(\mu ,\lambda \right)}\left(p_{XY}\right)$.*
*(b)*  *For any R,Δ≥0, we have*
  $$F\left(R,\Delta |p_{XY}\right)\geq \tilde{F}\left(R,\Delta |p_{XY}\right).$$
*(c)*  *Fix any $p=p_{UXY}\in P_{\mathrm{sh}}\left(p_{XY}\right)$ and μ∈[0,1]. For λ∈[0,1], ${\tilde{\Omega }}^{\left(\mu ,\lambda \right)}\left(p\right)$ exists and is nonnegative. For p=$p_{UXYZ}\in P_{\mathrm{sh}}\left(p_{XY}\right)$, define a probability distribution $p^{\left(\lambda \right)}=p_{UXYZ}^{\left(\lambda \right)}$ by*
  $$\begin{array}{ccc} p^{\left(\lambda \right)}\left(u,x,y,z\right) & := & \frac{p\left(u,x,y,z\right)\exp\left\{-\lambda {\tilde{\omega }}_{p}^{\left(\mu \right)}\left(x,y,z|u\right)\right\}}{E_{p}\left[\exp\left\{-\lambda {\tilde{\omega }}_{p}^{\left(\mu \right)}\left(X,Y,Z|U\right)\right\}\right]}. \end{array}$$
*Then, for λ∈[0,1/2], ${\tilde{\Omega }}^{\left(\mu ,\lambda \right)}\left(p\right)$ is twice differentiable. Furthermore, for λ∈[0,1/2], we have*
  $$\begin{array}{c} \frac{d}{d\lambda }{\tilde{\Omega }}^{\left(\mu ,\lambda \right)}\left(p\right)=E_{p^{\left(\lambda \right)}}\left[\omega_{p}^{\left(\mu \right)}\left(X,Y,Z|U\right)\right],\\ \frac{d^{2}}{d\lambda^{2}}{\tilde{\Omega }}^{\left(\mu ,\lambda \right)}\left(p\right)=-{\mathrm{Var}}_{p^{\left(\lambda \right)}}\left[{\tilde{\omega }}_{p}^{\left(\mu \right)}\left(X,Y,Z|U\right)\right]. \end{array}$$
*The second equality implies that ${\tilde{\Omega }}^{\left(\mu ,\lambda \right)}\left(p\right)$ is a concave function of λ∈[0,1/2].*
*(d)*  *For (μ,λ)∈[0,1]×[0,1/2], define*
  ρ(μ,λ)(pXY):=max(ν,p)∈[0,λ]×Psh(pXY):Ω˜(μ,λ)(p)=Ω˜(μ,λ)(pXY)Varp(ν)ω˜p(μ)(X,Y,Z|U),
  *and set*
  $$\begin{array}{c} \rho =\rho \left(p_{XY}\right):=\max_{\left(\mu ,\lambda )\in [0,1]\times [0,1/2\right]}\rho^{\left(\mu ,\lambda \right)}\left(p_{XY}\right). \end{array}$$
*Then, we have $\rho (p_{XY})<\infty$. Furthermore, for any (μ,λ)∈[0,1]×[0,1/2],*
  (9) $${\tilde{\Omega }}^{\left(\mu ,\lambda \right)}\left(p_{XY}\right)\geq \lambda R^{\left(\mu \right)}\left(p_{XY}\right)-\frac{\lambda^{2}}{2}\rho \left(p_{XY}\right).$$
*(e)*  *For every $\tau \in (0,\left(1/2\right)\rho \left(p_{XY}\right))$, the condition $\left(R+\tau ,\Delta +\tau \right)\notin R\left(p_{XY}\right)$ implies*
  $$\tilde{F}\left(R,\Delta |p_{XY}\right)>\frac{\rho (p_{XY})}{10}\cdot g^{2}\left(\frac{\tau }{\rho (p_{XY})}\right)>0,$$
  *where g is the inverse function of $\vartheta \left(a\right):=a+\left(1/5\right)a^{2},a\geq 0$.*

Proof of Property 4 Part (a) is given in Appendix B. Proof of Property 4 Part (b) is given in Appendix E. Proofs of Property 4 Parts (c), (d), and (e) are given in Appendix F.

Our main result is the following:

**Theorem** **3.** *For any R,Δ≥0, any $p_{XY}$, and for any $(\varphi^{\left(n\right)},$$\psi^{\left(n\right)})$ satisfying $\left(1/n\right)\log||\varphi^{\left(n\right)}\left|\right|\leq R,$ we have*

(10) $$P_{c}^{\left(n\right)}\left(\varphi^{\left(n\right)},\psi^{\left(n\right)};\Delta \right)\leq 5\exp\left\{-nF(R,\Delta |p_{XY})\right\}.$$

It follows from Theorem 3 and Property 4 Part (d) that if (R,Δ) is outside the rate distortion region, then the error probability of decoding goes to one exponentially and its exponent is not below $F(R,\Delta |p_{XY})$.

It immediately follows from Theorem 3 that we have the following corollary.

**Corollary** **1.** *For any R,Δ≥0 and any $p_{XY}$, we have*

(11) $$\begin{array}{c} G\left(R,\Delta |p_{XY}\right)\geq F\left(R,\Delta |p_{XY}\right). \end{array}$$

*Furthermore, for any $p_{XY}$, we have*

(12) $$\begin{array}{c} G\left(p_{XY}\right)\subseteq \bar{G}\left(p_{XY}\right):=\left\{\left(R,\Delta ,G\right):G\geq F\left(R,\Delta |p_{XY}\right)\right\}. \end{array}$$

Proof of Theorem 3 will be given in the next section. The exponent function in the case of Δ=0 can be obtained as a corollary of the result of Oohama and Han [9] for the separate source coding problem of correlated sources [10]. The techniques used by them is a method of types [4], which is not useful for proving Theorem 3. In fact, when we use this method, it is very hard to extract a condition related to the Markov chain condition U↔X↔Y, which the auxiliary random variable U∈U must satisfy when (R,Δ) is on the boundary of the set $R(p_{XY})$. Some novel techniques based on the information spectrum method introduced by Han [11] are necessary to prove this theorem.

From Theorem 3 and Property 4 Part (e), we can obtain an explicit outer bound of $R_{\mathrm{WZ}}\left(\varepsilon |p_{XY}\right)$ with an asymptotically vanishing deviation from $R_{\mathrm{WZ}}\left(p_{XY}\right)$ $=R(p_{XY})$. The strong converse theorem immediately follows from this corollary. To describe this outer bound, for κ>0, we set

$$\begin{array}{ccc} R\left(p_{XY}\right)-\kappa \left(1,1\right) & := & \{\left(R-\kappa ,\Delta -\kappa \right):\left(R,\Delta \right)\in R\left(p_{XY}\right)\}, \end{array}$$

which serves as an outer bound of $R(p_{XY})$. For each fixed ε∈(0,1), we define $\kappa_{n}$$=\kappa_{n}\left(\varepsilon ,\rho (p_{XY})\right)$ by

(13) $$\begin{array}{ccc} \kappa_{n} & := & \rho \left(p_{XY}\right)\vartheta \left(\sqrt{\frac{10}{n\rho (p_{XY})}\log\left(\frac{5}{1-\varepsilon }\right)}\right)\\ & \overset{\left(a\right)}{=} & \sqrt{\frac{10\rho (p_{XY})}{n}\log\left(\frac{5}{1-\varepsilon }\right)}+\frac{2}{n}\log\left(\frac{5}{1-\varepsilon }\right). \end{array}$$

Step (a) follows from $\vartheta \left(a\right)=a+\left(1/5\right)a^{2}$. Since $\kappa_{n}\to 0$ as n→∞, we have the smallest positive integer $n_{0}=n_{0}\left(\varepsilon ,\rho \left(p_{XY}\right)\right)$ such that $\kappa_{n}\leq \left(1/2\right)\rho \left(p_{XY}\right)$ for $n\geq n_{0}$. From Theorem 3 and Property 4 Part (e), we have the following corollary.

**Corollary** **2.** *For each fixed ε∈(0,1), we choose the above positive integer $n_{0}=$$n_{0}\left(\varepsilon ,\rho \left(p_{XY}\right)\right)$ Then, for any $n\geq n_{0}$, we have*

$$\begin{array}{c} R_{\mathrm{WZ}}\left(n,\varepsilon |p_{XY}\right)\subseteq R\left(p_{XY}\right)-\kappa_{n}\left(1,1\right). \end{array}$$

*The above result together with*

$$\begin{array}{ccc} R_{\mathrm{WZ}}\left(\varepsilon |p_{XY}\right) & = & \mathrm{cl}\left(\underset{m\geq 1}{⋃}\underset{n\geq m}{⋂}R_{\mathrm{WZ}}\left(n,\varepsilon |p_{XY}\right)\right), \end{array}$$

*yields that for each fixed ε∈(0,1), we have*

$$\begin{array}{c} R_{\mathrm{WZ}}\left(\varepsilon |p_{XY}\right)=R_{\mathrm{WZ}}\left(p_{XY}\right)=R\left(p_{XY}\right). \end{array}$$

Proof of this corollary will be given in the next section.

The direct part of coding theorem, i.e., the inclusion of $R(p_{XY})$⊆$R_{\mathrm{WZ}}\left(\varepsilon |p_{XY}\right)$ was established by Csiszár and Körner [4]. They proved a weak converse theorem to obtain the inclusion $R_{\mathrm{WZ}}\left(p_{XY}\right)$ $\subseteq R(p_{XY})$. Until now, we have had no result on the strong converse theorem. The above corollary stating the strong converse theorem for the Wyner–Ziv source coding problem implies that a long standing open problem since Csiszár and Körner [4] has been resolved.

## 4. Proof of the Main Results

In this section, we prove Theorem 3 and Corollary 2. We first present a lemma which upper bounds the correct probability of decoding by the information spectrum quantities. We set

$$S_{n}:=\varphi^{\left(n\right)}\left(X^{n}\right),Z^{n}:=\psi_{n}\left(\varphi^{\left(n\right)}\left(X^{n}\right),Y^{n}\right).$$

It is obvious that

$$S_{n}↔X^{n}↔Y^{n},X^{n}↔\left(S_{n},Y^{n}\right)↔Z^{n}.$$

Then, we have the following:

**Lemma** **1.** *For any η>0 and for any $(\varphi^{\left(n\right)}$, $\psi^{\left(n\right)})$ satisfying $\left(1/n\right)\log||\varphi^{\left(n\right)}\left|\right|\leq R,$ we have*

$$\begin{array}{c} \;P_{c}^{\left(n\right)}\left(\varphi^{\left(n\right)},\psi^{\left(n\right)};\Delta \right)\leq p_{S_{n}X^{n}Y^{n}Z^{n}}\{ \end{array}$$

(14) $$\begin{array}{ccc} \eta & \geq & \frac{1}{n}\log\frac{Q_{X^{n}}^{\left(i\right)}\left(X^{n}\right)}{p_{X^{n}}\left(X^{n}\right)}, \end{array}$$

(15) $$\begin{array}{ccc} \eta & \geq & \frac{1}{n}\log\frac{Q_{Y^{n}|S_{n}X^{n}}^{\left(\mathrm{ii}\right)}\left(Y^{n}|S_{n},X^{n}\right)}{p_{Y^{n}|X^{n}}\left(Y^{n}|X^{n}\right)}, \end{array}$$

(16) $$\begin{array}{ccc} \eta & \geq & \frac{1}{n}\log\frac{Q_{X^{n}|S_{n}Y^{n}Z^{n}}^{\left(\mathrm{iii}\right)}\left(X^{n}|S_{n},Y^{n},Z^{n}\right)}{p_{X^{n}|S_{n}Y^{n}}\left(X^{n}|S_{n},Y^{n}\right)}, \end{array}$$

(17) $$\begin{array}{ccc} R+\eta & \geq & \frac{1}{n}\log\frac{Q_{X^{n}|S_{n}Y^{n}}^{\left(\mathrm{iv}\right)}\left(X^{n}|S_{n},Y^{n}\right)}{p_{X^{n}|Y^{n}}\left(X^{n}|Y^{n}\right)}, \end{array}$$

(18) $$\begin{array}{ccc} \Delta & \geq & \left.\frac{1}{n}\log\exp\left\{d(X^{n},Z^{n})\right\}\right\}+4e^{-n\eta }. \end{array}$$

*The probability distribution and stochastic matrices appearing in the right members of Equation (18) have a property that we can select them arbitrary. In Equation (14), we can choose any probability distribution $Q_{X^{n}}^{\left(i\right)}$ on $X^{n}$. In Equation (15), we can choose any stochastic matrix $Q_{Y^{n}|S_{n}X^{n}}^{\left(\mathrm{ii}\right)}:$$M_{n}\times$$X^{n}$$\to Y^{n}$. In Equation (16), we can choose any stochastic matrix $Q_{X^{n}|S_{n}Y^{n}Z^{n}}^{\left(\mathrm{iii}\right)}:$$M_{n}\times$$Y^{n}$×$Z^{n}$$\to X^{n}$. In Equation (17), we can choose any stochastic matrix $Q_{X^{n}|S_{n}Y^{n}}^{\left(\mathrm{iv}\right)}:$$M_{n}$×$Y^{n}$$\to X^{n}$.*

Proof of this lemma is given in Appendix G.

**Lemma** **2.** *Suppose that, for each t=1,2,⋯,n, the joint distribution $p_{S_{n}X^{t}Y^{n}}$ of the random vector $S_{n}X^{t}Y^{n}$ is a marginal distribution of $p_{S_{n}X^{n}Y^{n}}$. Then, for t=1,2,⋯,n, we have the following Markov chain:*

(19) $$X_{t}↔S_{n}X^{t-1}Y_{t}^{n}↔Y^{t-1}$$

*or equivalently that $I(X_{t};Y^{t-1}|S_{n}X^{t-1}Y_{t}^{n})=0$.*

Proof of this lemma is given in Appendix H. For t=1,2,⋯,n, set $u_{t}:=\left(s,x^{t-1},y_{t+1}^{n}\right)$. Let $U_{t}:=$$(S_{n},X^{t-1},$$Y_{t+1}^{n})$ be a random vector taking values in $M_{n}$ ×$X^{t-1}$ ×$Y_{t+1}^{n}$. From Lemmas 1 and 2, we have the following:

**Lemma** **3.** *For any η>0 and for any $(\varphi^{\left(n\right)}$, $\psi^{\left(n\right)})$ satisfying $\left(1/n\right)\log||\varphi^{\left(n\right)}\left|\right|\leq R,$ we have the following:*

(20) $$\begin{array}{c} P_{c}^{\left(n\right)}\left(\varphi^{\left(n\right)},\psi^{\left(n\right)};\Delta \right)\leq p_{S_{n}X^{n}Y^{n}Z^{n}}\{\\ \begin{array}{ccc} \eta & \geq & \frac{1}{n}\sum_{t=1}^{n}\log\frac{Q_{X_{t}}^{\left(i\right)}\left(X_{t}\right)}{p_{X_{t}}\left(X_{t}\right)},\\ \eta & \geq & \frac{1}{n}\sum_{t=1}^{n}\log\frac{Q_{Y_{t}|U_{t}X_{t}}^{\left(\mathrm{ii}\right)}\left(Y_{t}|U_{t},X_{t}\right)}{p_{Y_{t}|X_{t}}\left(Y_{t}|X_{t}\right)},\\ \eta & \geq & \frac{1}{n}\sum_{t=1}^{n}\log\frac{Q_{X_{t}|U_{t}Y_{t}Z_{t}}^{\left(\mathrm{iii}\right)}\left(X_{t}|U_{t},Y_{t},Z_{t}\right)}{p_{X_{t}|U_{t}Y_{t}}\left(X_{t}|U_{t},Y_{t}\right)},\\ R+\eta & \geq & \frac{1}{n}\sum_{t=1}^{n}\log\frac{Q_{X_{t}|U_{t}Y_{t}}^{\left(\mathrm{iv}\right)}\left(X_{t}|U_{t},Y_{t}\right)}{p_{X_{t}|Y_{t}}\left(X_{t}|Y_{t}\right)},\\ \Delta & \geq & \left.\frac{1}{n}\sum_{t=1}^{n}\log e^{d(X_{t},Z_{t})}\right\}+4e^{-n\eta }, \end{array} \end{array}$$

*where for each t=1,2,⋯,n, the following probability distribution and stochastic matrices:*

$$Q_{X_{t}}^{\left(i\right)},Q_{Y_{t}|U_{t}X_{t}}^{\left(\mathrm{ii}\right)},Q_{X_{t}|U_{t}Y_{t}Z_{t}}^{\left(\mathrm{iii}\right)},\;and\;Q_{X_{t}|U_{t}Y_{t}}^{\left(\mathrm{iv}\right)}$$

*appearing in the first term in the right members of Equation (21) have a property that we can choose their values arbitrary.*

**Proof.**  On the probability distributions appearing in the right members of Equation (18), we take the following choices. In Equation (14), we choose $Q_{X^{n}}^{\left(i\right)}$ so that

(21) $$Q_{X^{n}}^{\left(i\right)}\left(X^{n}\right)=\prod_{t=1}^{n}Q_{X_{t}}^{\left(i\right)}\left(X_{t}\right).$$

In Equation (15), we choose $Q_{Y^{n}|S_{n}X^{n}}^{\left(\mathrm{ii}\right)}$ so that

(22) $$\begin{array}{ccc} Q_{Y^{n}|S_{n}X^{n}}^{\left(\mathrm{ii}\right)}\left(Y^{n}|S_{n},X^{n}\right) & = & \prod_{t=1}^{n}Q_{Y_{t}|S_{n}X^{t}Y_{t+1}^{n}}^{\left(\mathrm{ii}\right)}\left(Y_{t}|S_{n},X^{t},Y_{t+1}^{n}\right)=\prod_{t=1}^{n}Q_{Y_{t}|X_{t}U_{t}}^{\left(\mathrm{ii}\right)}\left(Y_{t}|U_{t}X_{t}\right). \end{array}$$

In Equation (16), we choose $Q_{X^{n}|S_{n}Y^{n}Z^{n}}^{\left(\mathrm{iii}\right)}$ so that

(23) $$\begin{array}{ccc} Q_{X^{n}|S_{n}Y^{n}Z^{n}}^{\left(\mathrm{iii}\right)}\left(X^{n}|S_{n},Y^{n},Z^{n}\right) & = & \prod_{t=1}^{n}Q_{X_{t}|S_{n}X^{t-1}Y_{t}^{n}Z_{t}}^{\left(\mathrm{iii}\right)}\left(X_{t}|S_{n}X^{t-1},Y_{t}^{n},Z_{t}\right)\\ & = & \prod_{t=1}^{n}Q_{X_{t}|U_{t}Y_{t}Z_{t}}^{\left(\mathrm{iii}\right)}\left(X_{t}|U_{t}Y_{t}Z_{t}\right). \end{array}$$

In Equation (16), we note that

(24) $$\begin{array}{ccc} p_{X^{n}|S_{n}Y^{n}}\left(X^{n}|S_{n},Y^{n}\right) & = & \prod_{t=1}^{n}p_{X_{t}|S_{n}X^{t-1}Y^{n}}\left(X_{t}|S_{n},X^{t-1},Y^{n}\right)\overset{\left(a\right)}{=}\prod_{t=1}^{n}p_{X_{t}|S_{n}X^{t-1}Y_{t}^{n}}\left(X_{t}|S_{n},X^{t-1},Y_{t}^{n}\right)\\ & = & \prod_{t=1}^{n}p_{X_{t}|U_{t}Y_{t}}\left(X_{t}|U_{t},Y_{t}\right). \end{array}$$

Step (a) follows from Lemma 2. In Equation (17), we choose $Q_{X^{n}|S_{n}Y^{n}}^{\left(\mathrm{iv}\right)}$ so that

(25) $$\begin{array}{c} Q_{X^{n}|S_{n}Y^{n}}^{\left(\mathrm{iv}\right)}\left(X^{n}|S_{n},Y^{n}\right)=\prod_{t=1}^{n}Q_{X_{t}|S_{n}X^{t-1}Y_{t}^{n}}^{\left(\mathrm{iv}\right)}\left(X_{t}|S_{n},X^{t-1},Y_{t}^{n}\right)=\prod_{t=1}^{n}Q_{X_{t}|U_{t}Y_{t}}^{\left(\mathrm{iv}\right)}\left(X_{t}|U_{t},Y_{t}\right). \end{array}$$

From Lemma 1 and Equations (21)–(25), we have the bound of Equation (21) in Lemma 3. ☐

To evaluate an upper btound of Equation (21) in Lemma 3, we use the following lemma, which is well known as the Cramér’s bound in the large deviation principle.

**Lemma** **4.** *For any real valued random variable A and any θ≥0, we have*

$$\mathrm{Pr}\left\{A\leq a\right\}\leq \exp\left[\left(\theta a+\log E[\exp(-\theta A)]\right)\right].$$

Here, we define a quantity which serves as an exponential upper bound of $P_{c}^{\left(n\right)}(\varphi^{\left(n\right)},$ $\psi^{\left(n\right)})$. For each t=1,2,⋯,n, let ${\underline{Q}}_{t}$ be a set of all

$$\begin{array}{ccc} {\underline{Q}}_{t} & = & (Q_{X_{t}}^{\left(i\right)},Q_{Y_{t}|U_{t}X_{t}}^{\left(\mathrm{ii}\right)},Q_{X_{t}|U_{t}Y_{t}Z_{t}}^{\left(\mathrm{iii}\right)},Q_{X_{t}|U_{t}Y_{t}}^{\left(\mathrm{iv}\right)}). \end{array}$$

Set

$$\begin{array}{ccc} {\underline{Q}}^{n} & := & \prod_{t=1}^{n}{\underline{Q}}_{t},{\underline{Q}}^{n}:={\left\{{\underline{Q}}_{t}\right\}}_{t=1}^{n}\in {\underline{Q}}^{n}. \end{array}$$

Let $P^{\left(n\right)}\left(p_{XY}\right)$ be a set of all probability distributions $p_{S_{n}X^{n}Y^{n}Z^{n}}$ on $M_{n}\times X^{n}\times Y^{n}\times Z^{n}$ having the form:

$$\begin{array}{ccc} p_{S_{n}X^{n}Y^{n}Z^{n}}\left(s,x^{n},y^{n},z^{n}\right) & = & p_{S_{n}|X^{n}}\left(s|x^{n}\right)\left\{\prod_{t=1}^{n}p_{X_{t}Y_{t}}\left(x_{t},y_{t}\right)\right\}p_{Z^{n}|Y^{n}S_{n}}\left(z^{n}|y^{n},s\right). \end{array}$$

For simplicity of notation, we use the notation $p^{\left(n\right)}$ for $p_{S_{n}X^{n}Y^{n}Z^{n}}$$\in P^{\left(n\right)}$ $\left(p_{XY}\right)$. We assume that $p_{U_{t}X_{t}Y_{t}Z_{t}}=p_{S_{n}X^{t}Y_{t}^{n}Z_{t}}$ is a marginal distribution of $p^{\left(n\right)}$. For t=1,2,⋯,n, we simply write $p_{t}=$$p_{U_{t}X_{t}Y_{t}Z_{t}}$. For $p^{\left(n\right)}$ $\in P^{\left(n\right)}\left(p_{XY}\right)$ and ${\underline{Q}}^{n}$$\in {\underline{Q}}^{n}$, we define

$$\begin{array}{c} \Omega_{}^{\left(\mu ,\lambda ,\theta \right)}\left(p^{\left(n\right)},{\underline{Q}}^{n}|p_{XY}\right):=-\log E_{p^{\left(n\right)}}\left[\left(\prod_{t=1}^{n}\left\{\frac{p_{X_{t}}\left(X_{t}\right)}{Q_{X_{t}}^{\left(i\right)}\left(X_{t}\right)}\right.\right.\right.\left.{\left.\frac{p_{Y_{t}|X_{t}}\left(Y_{t}|X_{t}\right)}{Q_{Y_{t}|X_{t}U_{t}}^{\left(\mathrm{ii}\right)}\left(Y_{t}|X_{t},U_{t}\right)}\right\}}^{\theta }\right)\\ \times \left(\prod_{t=1}^{n}{\left\{\frac{p_{X_{t}|U_{t}Y_{t}}\left(X_{t}|U_{t},Y_{t}\right)}{Q_{X_{t}|U_{t}Y_{t}Z_{t}}^{\left(\mathrm{iii}\right)}\left(X_{t}|U_{t},Y_{t},Z_{t}\right)}\right\}}^{\theta }\right)\left.\left(\prod_{t=1}^{n}{\left\{{\left(\frac{p_{X_{t}|Y_{t}}\left(X_{t}|Y_{t}\right)}{Q_{X_{t}|Y_{t}U_{t}}^{\left(\mathrm{iv}\right)}\left(X_{t}|U_{t},Y_{t}\right)}\right)}^{\bar{\mu }}e^{-\mu d(X_{t},Z_{t})}\right\}}^{\lambda \theta }\right)\right], \end{array}$$

where, for each t=1,2,⋯,n, the following probability distribution and stochastic matrices:

$$Q_{X_{t}}^{\left(i\right)},Q_{X_{t}|U_{t}Y_{t}}^{\left(\mathrm{ii}\right)},Q_{X_{t}|U_{t}Y_{t}Z_{t}}^{\left(\mathrm{iii}\right)},Q_{Y_{t}|X_{t}U_{t}}^{\left(\mathrm{iv}\right)}$$

appearing in the definition of $\Omega_{}^{\left(\mu ,\lambda ,\theta \right)}\left(p^{\left(n\right)},{\underline{Q}}^{n}|p_{XY}\right)$ are chosen so that they are induced by the joint distribution $q_{t}=q_{U_{t}X_{t}Y_{t}Z_{t}}\in Q_{t}$.

By Lemmas 3 and 4, we have the following proposition:

**Proposition** **1.** *For any μ∈[0,1],λ,θ≥0, any $q^{n}\in Q^{n}$, and any $(\varphi^{\left(n\right)},$$\psi^{\left(n\right)})$ satisfying $\left(1/n\right)\log||\varphi^{\left(n\right)}\left|\right|\leq R,$ we have*

$$\begin{array}{ccc} P_{c}^{\left(n\right)}\left(\varphi^{\left(n\right)},\psi^{\left(n\right)};\Delta \right) & \leq & 5\exp\{-n{\left[1+(3+\lambda \bar{\mu })\theta \right]}^{-1}\left.\left[\frac{1}{n}\Omega_{}^{\left(\mu ,\lambda ,\theta \right)}\left(p^{\left(n\right)},{\underline{Q}}^{n}|p_{XY}\right)-\lambda \theta \left(\bar{\mu }R+\mu \Delta \right)\right]\right\}. \end{array}$$

**Proof.**  When $\Omega_{}^{\left(\mu ,\lambda ,\theta \right)}\left(p^{\left(n\right)},{\underline{Q}}^{n}|p_{XY}\right)\leq n\lambda \theta \left(\bar{\mu }R+\mu \Delta \right)$, the bound we wish to prove is obvious. In the following argument, we assume that $\Omega_{}^{\left(\mu ,\lambda ,\theta \right)}\left(p^{\left(n\right)},{\underline{Q}}^{n}|p_{XY}\right)>$ $n\lambda \theta (\bar{\mu }R+\mu \Delta ).$ We define five random variables $A_{i},$i=1,2,⋯,5 by

$$\begin{array}{c} A_{1}=\frac{1}{n}\sum_{t=1}^{n}\log\frac{Q_{X_{t}}^{\left(i\right)}\left(X_{t}\right)}{p_{X_{t}}\left(X_{t}\right)},A_{2}=\frac{1}{n}\sum_{t=1}^{n}\log\frac{Q_{Y_{t}|X_{t}U_{t}}^{\left(\mathrm{ii}\right)}\left(Y_{t}|X_{t},U_{t}\right)}{p_{Y_{t}|X_{t}}\left(Y_{t}|X_{t}\right)},\\ A_{3}=\frac{1}{n}\sum_{t=1}^{n}\log\frac{Q_{X_{t}|U_{t}Y_{t}Z_{t}}^{\left(\mathrm{iii}\right)}\left(X_{t}|U_{t},Y_{t},Z_{t}\right)}{p_{X_{t}|U_{t}Y_{t}}\left(X_{t}|U_{t},Y_{t}\right)},\\ A_{4}=\frac{1}{n}\sum_{t=1}^{n}\log\frac{Q_{X_{t}|U_{t}Y_{t}}^{\left(\mathrm{iv}\right)}\left(X_{t}|U_{t},Y_{t}\right)}{p_{X_{t}|Y_{t}}\left(X_{t}|Y_{t}\right)},A_{5}=\frac{1}{n}\sum_{t=1}^{n}\log e^{d(X_{t},Z_{t})}. \end{array}$$

By Lemma 3, for any $\left(\varphi^{\left(n\right)},\psi^{\left(n\right)}\right)$ satisfying $\left(1/n\right)\log||\varphi^{\left(n\right)}\left|\right|$≤R, we have

(26) $$\begin{array}{c} P_{c}^{\left(n\right)}\left(\varphi^{\left(n\right)},\psi^{\left(n\right)};\Delta \right)\leq p_{S_{n}X^{n}Y^{n}Z^{n}}\left\{A_{i}\leq \eta fori=1,2,3,A_{4}\leq R+\eta ,A_{5}\leq \Delta \right\}+4e^{-n\eta }\\ \leq p_{S_{n}X^{n}Y^{n}Z^{n}}\left\{A_{1}+A_{2}+A_{3}+\lambda \left(\bar{\mu }A_{4}+\mu A_{5}\right)\leq \lambda \left(\bar{\mu }R+\mu \Delta \right)+\left(3+\lambda \bar{\mu }\right)\eta \right\}+4e^{-n\eta }\\ =p_{S_{n}X^{n}Y^{n}Z^{n}}\left\{A\leq a\right\}+4e^{-n\eta }, \end{array}$$

where we set

$$\begin{array}{ccc} A & := & A_{1}+A_{2}+A_{3}+\lambda \left(\bar{\mu }A_{4}+\mu A_{5}\right),\\ a & := & \lambda \left(\bar{\mu }R+\mu \Delta \right)+\left(3+\lambda \bar{\mu }\right)\eta . \end{array}$$

Applying Lemma 4 to the first term in the right member of Equation (26), we have

(27) $$\begin{array}{cc} & P_{c}^{\left(n\right)}\left(\varphi^{\left(n\right)},\psi^{\left(n\right)};\Delta \right)\leq \exp\left[\left(\theta a+\log E_{p^{\left(n\right)}}\left[\exp\left(-\theta A\right)\right]\right)\right]+4e^{-n\eta }\\ = & \exp[n\{\lambda \theta \left(\bar{\mu }R+\mu \Delta \right)+\left(3+\lambda \bar{\mu }\right)\theta \eta \left.\left.-\frac{1}{n}\Omega_{}^{\left(\mu ,\lambda ,\theta \right)}\left(p^{\left(n\right)},{\underline{Q}}^{n}|p_{XY}\right)\right\}\right]+4e^{-n\eta }. \end{array}$$

We choose η so that

(28) $$\begin{array}{ccc} -\eta & = & \lambda \theta \left(\bar{\mu }R+\mu \Delta \right)+\theta \left(3+\lambda \bar{\mu }\right)\eta -\frac{1}{n}\Omega_{}^{\left(\mu ,\lambda ,\theta \right)}\left(p^{\left(n\right)},{\underline{Q}}^{n}|p_{XY}\right). \end{array}$$

Solving Equation (28) with respect to η, we have

$$\begin{array}{c} \eta =\frac{\frac{1}{n}\Omega_{}^{\left(\mu ,\lambda ,\theta \right)}\left(p^{\left(n\right)},{\underline{Q}}^{n}|p_{XY}\right)-\lambda \theta \left(\bar{\mu }R+\mu \Delta \right)}{1+(3+\lambda \bar{\mu })\theta }. \end{array}$$

For this choice of η and Equation (27), we have

$$\begin{array}{cc} & P_{c}^{\left(n\right)}\left(\varphi^{\left(n\right)},\psi^{\left(n\right)};\Delta \right)\leq 5e^{-n\eta }\\ = & 5\exp\{-n{\left[1+(3+\lambda \bar{\mu })\theta \right]}^{-1}\left.\left[\frac{1}{n}\Omega_{}^{\left(\mu ,\lambda ,\theta \right)}\left(p^{\left(n\right)},{\underline{Q}}^{n}|p_{XY}\right)-\lambda \theta \left(\bar{\mu }R+\mu \Delta \right)\right]\right\}, \end{array}$$

completing the proof. ☐

Set

$$\begin{array}{ccc} {\underline{\Omega }}^{\left(\mu ,\lambda ,\theta \right)}\left(p_{XY}\right) & := & \underset{n\geq 1}{\inf}\min_{p^{\left(n\right)}\in P^{\left(n\right)}\left(p_{XY}\right)}\max_{{\underline{Q}}^{n}\in {\underline{Q}}^{n}}\frac{1}{n}\Omega_{}^{\left(\mu ,\lambda ,\theta \right)}\left(p^{\left(n\right)},{\underline{Q}}^{n}|p_{XY}\right). \end{array}$$

By Proposition 1, we have the following corollary.

**Corollary** **3.** *For any μ∈[0,1],λ≥0, for any θ≥0, and for any $(\varphi^{\left(n\right)},$$\psi^{\left(n\right)})$ satisfying $\left(1/n\right)\log||\varphi^{\left(n\right)}\left|\right|\leq R,$ we have*

$$\begin{array}{ccc} P_{c}^{\left(n\right)}\left(\varphi^{\left(n\right)},\psi^{\left(n\right)};\Delta \right) & \leq & 5\exp\left\{-n\left[\frac{{\underline{\Omega }}^{\left(\mu ,\lambda ,\theta \right)}\left(p_{XY}\right)-\lambda \theta \left(\bar{\mu }R+\mu \Delta \right)}{1+(3+\lambda \bar{\mu })\theta }\right]\right\}. \end{array}$$

We shall call ${\underline{\Omega }}^{\left(\mu ,\lambda ,\theta \right)}\left(p_{XY}\right)$ the communication potential. The above corollary implies that the analysis of ${\underline{\Omega }}^{\left(\mu ,\lambda ,\theta \right)}\left(p_{XY}\right)$ leads to an establishment of a strong converse theorem for Wyner–Ziv source coding problem. In the following argument, we drive an explicit lower bound of ${\underline{\Omega }}^{\left(\mu ,\lambda ,\theta \right)}\left(p_{XY}\right)$. We use a new technique we call *the recursive method*. The recursive method is a powerful tool to drive a single letterized exponent function for rates below the rate distortion function. This method is also applicable to prove the exponential strong converse theorems for other network information theory problems [5,6,7]. Set

$$F_{t}:=\left(p_{X_{t}|U_{t}Y_{t}},{\underline{Q}}_{t}\right),\;F^{t}:={\left\{F_{i}\right\}}_{i=1}^{t}.$$

For each t=1,2,⋯,n, define a function of $\left(u_{t},x_{t},y_{t},z_{t}\right)$ $\in U_{t}$ ×X ×Y ×Z by

$$\begin{array}{c} f_{F_{t}}^{\left(\mu ,\lambda ,\theta \right)}\left(x_{t},y_{t},z_{t}|u_{t}\right)\\ :=\left\{\frac{p_{X_{t}}\left(x_{t}\right)}{Q_{X_{t}}^{\left(i\right)}\left(x_{t}\right)}\frac{p_{Y_{t}|X_{t}}\left(y_{t}|x_{t}\right)}{Q_{Y_{t}|X_{t}U_{t}}^{\left(\mathrm{ii}\right)}\left(y_{t}|x_{t},u_{t}\right)}\right.{\left.\frac{p_{X_{t}|U_{t}Y_{t}}\left(x_{t}|u_{t},y_{t}\right)}{Q_{X_{t}|U_{t}Y_{t}Z_{t}}^{\left(\mathrm{iii}\right)}\left(x_{t}|u_{t},y_{t},z_{t}\right)}{\left(\frac{p_{X_{t}|Y_{t}}\left(x_{t}|y_{t}\right)}{Q_{X_{t}|Y_{t}U_{t}}^{\left(\mathrm{iv}\right)}\left(x_{t}|u_{t},y_{t}\right)}\right)}^{\lambda \bar{\mu }}e^{-\lambda \mu d(x_{t},z_{t})}\right\}}^{\theta }. \end{array}$$

By definition, we have

(29) $$\begin{array}{cc} & \exp\left\{-\Omega_{}^{\left(\mu ,\lambda ,\theta \right)}\left(p^{\left(n\right)},{\underline{Q}}^{n}|p_{XY}\right)\right\}\\ = & \sum_{s,y^{n}}p_{S_{n}Y^{n}}\left(s,y^{n}\right)\sum_{x^{n},z^{n}}p_{X^{n}Z^{n}|S_{n}Y^{n}}\left(x^{n},z^{n}|s,y^{n}\right)\prod_{t=1}^{n}f_{F_{t}}^{\left(\mu ,\lambda ,\theta \right)}\left(x_{t},y_{t},z_{t}|u_{t}\right). \end{array}$$

For each t=1,2,⋯,n, we define the conditional probability distribution

$$\begin{array}{ccc} p_{X^{t}Z^{t}|S_{n}Y^{n};F^{t}}^{\left(\mu ,\lambda ,\theta \right)} & := & {\left\{p_{X^{t}Z^{t}|S_{n}Y^{n};F^{t}}^{\left(\mu ,\lambda ,\theta \right)}\left(x^{t},z^{t}|s,y^{n}\right)\right\}}_{\left(x^{t},z^{t},s,y^{n}\right)\in X^{t}\times Z^{t}\times M_{n}\times Y^{n}} \end{array}$$

by

$$\begin{array}{ccc} p_{X^{t}Z^{t}|S_{n}Y^{n};F^{t}}^{\left(\mu ,\lambda ,\theta \right)}\left(x^{t},z^{t}|s,y^{n}\right) & := & C_{t}^{-1}\left(s,y^{n}\right)p_{X^{t}Z^{t}|S_{n}Y^{n}}\left(x^{t},z^{t}|s,y^{n}\right)\prod_{i=1}^{t}f_{F_{i}}^{\left(\mu ,\lambda ,\theta \right)}\left(x_{i},y_{i},z_{i}|u_{i}\right) \end{array}$$

where

(31) $$\begin{array}{ccc} C_{t}\left(s,y^{n}\right) & := & \sum_{x^{t},z^{t}}p_{X^{t}Z^{t}|S_{n}Y^{n}}\left(x^{t},z^{t}|s,y^{n}\right)\prod_{i=1}^{t}f_{F_{i}}^{\left(\mu ,\lambda ,\theta \right)}\left(x_{i},y_{i},z_{i}|u_{i}\right) \end{array}$$

are constants for normalization. For t=1,2,⋯,n, define

(31) $$\Phi_{t,F^{t}}^{\left(\mu ,\lambda ,\theta \right)}\left(s,y^{n}\right):=C_{t}\left(s,y^{n}\right)C_{t-1}^{-1}\left(s,y^{n}\right),$$

where we define $C_{0}\left(s,y^{n}\right)=1$ for $\left(s,y^{n}\right)\in M_{n}$$\times Y^{n}.$ Then, we have the following lemma:

**Lemma** **5.** *For each t=1,2,⋯,n, and for any (s,$y^{n}$$x^{t},z^{t})\in M_{n}$$\times Y^{n}$$\times X^{t}$$\times Z^{t}$, we have*

$$\begin{array}{c} p_{X^{t}Z^{t}|S_{n}Y^{n};F^{t}}^{\left(\mu ,\lambda ,\theta \right)}\left(x^{t},z^{t}|s,y^{n}\right)={\left(\Phi_{t,F^{t}}^{\left(\mu ,\lambda ,\theta \right)}\left(s,y^{n}\right)\right)}^{-1}p_{X^{t-1}Y^{t-1}|S_{n}Y^{n};F^{t-1}}^{\left(\mu ,\lambda ,\theta \right)}\left(x^{t-1},z^{t-1}|s,y^{n}\right) \end{array}$$

(32) $$\begin{array}{c} \;\times p_{X_{t}Z_{t}|S_{n}X^{t-1}Y^{n}}\left(x_{t},z_{t}|s,x^{t-1},z^{t-1},y^{n}\right)f_{F_{t}}^{\left(\mu ,\lambda ,\theta \right)}\left(x_{t},y_{t},z_{t}|u_{t}\right).\\ \Phi_{t,F^{t}}^{\left(\mu ,\lambda ,\theta \right)}\left(s,y^{n}\right)=\sum_{x^{t},z^{t}}p_{X^{t-1}Z^{t-1}|S_{n}Y^{n};F^{t-1}}^{\left(\mu ,\lambda ,\theta \right)}\left(x^{t-1},z^{t-1}|s,y^{n}\right) \end{array}$$

(33) $$\begin{array}{c} \;\times p_{X_{t}Z_{t}|S_{n}X^{t-1}Y^{n}}\left(x_{t},z_{t}|s,x^{t-1},z^{t-1},y^{n}\right)f_{F_{t}}^{\left(\mu ,\lambda ,\theta \right)}\left(x_{t},y_{t},z_{t}|u_{t}\right). \end{array}$$

*Furthermore, we have*

(34) $$\begin{array}{ccc} \exp\left\{-\Omega_{}^{\left(\mu ,\lambda ,\theta \right)}\left(p^{\left(n\right)},{\underline{Q}}^{n}|p_{XY}\right)\right\} & = & \sum_{s,y^{n}}p_{S_{n}Y^{n}}\left(s,y^{n}\right)\prod_{t=1}^{n}\Phi_{t,F^{t}}^{\left(\mu ,\lambda ,\theta \right)}\left(s,y^{n}\right). \end{array}$$

The equality in Equation (34) in Lemma 5 is obvious from Equations (29)–(31). Proofs of Equations (32) and (33) in this lemma are given in Appendix I. Next, we define a probability distribution of the random pair $\left(S_{n},Y^{n}\right)$ taking values in $M_{n}$$\times Y^{n}$ by

(35) $$\begin{array}{c} p_{S_{n}Y^{n};F^{t}}^{\left(\mu ,\lambda ,\theta \right)}\left(s,y^{n}\right)={\tilde{C}}_{t}^{-1}p_{S_{n}Y^{n}}\left(s,y^{n}\right)\prod_{i=1}^{t}\Phi_{i,F^{i}}^{\left(\mu ,\lambda ,\theta \right)}\left(s,y^{n}\right), \end{array}$$

where ${\tilde{C}}_{t}$ is a constant for normalization given by

$${\tilde{C}}_{t}=\sum_{s,y^{n}}p_{S_{n}Y^{n}}\left(s,y^{n}\right)\prod_{i=1}^{t}\Phi_{i,F^{i}}^{\left(\mu ,\lambda ,\theta \right)}\left(s,y^{n}\right).$$

For t=1,2,⋯,n, define

(36) $$\Lambda_{t,F^{t}}^{\left(\mu ,\lambda ,\theta \right)}:=\tilde{C_{t}}{\tilde{C}}_{t-1}^{-1},$$

where we define ${\tilde{C}}_{0}=1$. Set

(37) $$\begin{array}{c} p_{S_{n}X^{t}Y_{t}^{n}Z_{t};F^{t-1}}^{\left(\mu ,\lambda ,\theta \right)}\left(s,x^{t},y_{t}^{n},z_{t}\right)=p_{U_{t}X_{t}Y_{t}Z_{t};F^{t-1}}^{\left(\mu ,\lambda ,\theta \right)}\left(u_{t},x_{t},y_{t},z_{t}\right)\\ :=\sum_{y^{t-1},z^{t-1}}p_{S_{n}Y^{n};F^{t-1}}^{\left(\mu ,\lambda ,\theta \right)}\left(s,y^{n}\right)p_{X^{t-1}Z^{t-1}|S_{n}Y^{n};F^{t-1}}^{\left(\mu ,\lambda ,\theta \right)}\left(x^{t-1},z^{t-1}|s,y^{n}\right)\\ \;\times p_{X_{t}Z_{t}|X^{t-1}Z^{t-1}S_{n}Y^{n}}\left(x_{t},z_{t}|x^{t-1},z^{t-1},s,y^{n}\right). \end{array}$$

Then, we have the following:

**Lemma** **6.** (38) $$\begin{array}{c} \exp\left\{-\Omega_{}^{\left(\mu ,\lambda ,\theta \right)}\left(p^{\left(n\right)},{\underline{Q}}^{n}|p_{XY}\right)\right\}=\prod_{t=1}^{n}\Lambda_{t,F^{t}}^{\left(\mu ,\lambda ,\theta \right)},\; \end{array}$$

(39) $$\begin{array}{c} \Lambda_{t,F^{t}}^{\left(\mu ,\lambda ,\theta \right)}=\sum_{u_{t},x_{t},y_{t},z_{t}}p_{U_{t}X_{t}Y_{t}Z_{t};F^{t-1}}^{\left(\mu ,\lambda ,\theta \right)}\left(u_{t},x_{t},y_{t},z_{t}\right)f_{F_{t}}^{\left(\mu ,\lambda ,\theta \right)}\left(x_{t},y_{t},z_{t}|u_{t}\right). \end{array}$$

**Proof.**  By the equality Equation (34) in Lemma 5, we have

(40) $$\begin{array}{c} \exp\left\{-\Omega_{}^{\left(\mu ,\lambda ,\theta \right)}\left(p^{\left(n\right)},{\underline{Q}}^{n}|p_{XY}\right)\right\}={\tilde{C}}_{n}=\prod_{t=1}^{n}{\tilde{C}}_{t}{\tilde{C}}_{t-1}^{-1}\overset{\left(a\right)}{=}\prod_{t=1}^{n}\Lambda_{t,F^{t}}^{\left(\mu ,\lambda ,\theta \right)}. \end{array}$$

Step (a) follows from the definition in Equation (36) of $\Lambda_{t,F^{t}}^{\left(\mu ,\lambda ,\nu ,\theta \right)}.$ We next prove Equation (39) in Lemma 6. Multiplying $\Lambda_{t,F^{t}}^{\left(\mu ,\lambda ,\theta \right)}=$${\tilde{C}}_{t}/{\tilde{C}}_{t-1}$ to both sides of Equation (35), we have

(41) $$\begin{array}{cc} & \Lambda_{t,F^{t}}^{\left(\mu ,\lambda ,\theta \right)}p_{S_{n}Y^{n};F^{t}}^{\left(\mu ,\lambda ,\theta \right)}\left(s,y^{n}\right)\\ = & {\tilde{C}}_{t-1}^{-1}p_{S_{n}Y^{n}}\left(s,y^{n}\right)\prod_{i=1}^{t}\Phi_{i,F^{i}}^{\left(\mu ,\lambda ,\theta \right)}\left(s,y^{n}\right) \end{array}$$

(42) $$\begin{array}{cc} = & p_{S_{n}Y^{n};F^{t-1}}^{\left(\mu ,\lambda ,\theta \right)}\left(s,y^{n}\right)\Phi_{t,F^{t}}^{\left(\mu ,\lambda ,\theta \right)}\left(s,y^{n}\right). \end{array}$$

Taking summations of Equations (41) and (42) with respect to $\left(s,y^{n}\right)$, we have

$$\begin{array}{c} \Lambda_{t,F^{t}}^{\left(\mu ,\lambda ,\theta \right)}=\sum_{s,y^{n}}p_{S_{n}Y^{n};F^{t-1}}^{\left(\mu ,\lambda ,\theta \right)}\left(s,y^{n}\right)\Phi_{t,F^{t}}^{\left(\mu ,\lambda ,\theta \right)}\left(s,y^{n}\right)\\ \overset{\left(a\right)}{=}\sum_{s,y^{n}}p_{S_{n}Y^{n};F^{t-1}}^{\left(\mu ,\lambda ,\theta \right)}\left(s,y^{n}\right)\sum_{x^{t},z^{t}}p_{X^{t-1}Z^{t-1}|S_{n}Y^{n};F^{t-1}}^{\left(\mu ,\lambda ,\theta \right)}\left(x^{t-1},z^{t-1}|s,y^{n}\right)\\ \;\times p_{X_{t}Z_{t}|X^{t-1}Z^{t-1}S_{n}Y^{n}}\left(x_{t},z_{t}|x^{t-1},z^{t-1},s,y^{n}\right)f_{F_{t}}^{\left(\mu ,\lambda ,\theta \right)}\left(x_{t},y_{t},z_{t}|u_{t}\right)\\ =\sum_{s,x^{t},y_{t}^{n},z_{t}}\sum_{y^{t-1},z^{t-1}}p_{S_{n}Y^{n};F^{t-1}}^{\left(\mu ,\lambda ,\theta \right)}\left(s,y^{n}\right)p_{X^{t-1}Z^{t-1}|S_{n}Y^{n};F^{t-1}}^{\left(\mu ,\lambda ,\theta \right)}\left(x^{t-1},z^{t-1}|s,y^{n}\right)\\ \;\times p_{X_{t}Z_{t}|X^{t-1}Z^{t-1}S_{n}Y^{n}}\left(x_{t},z_{t}|x^{t-1},z^{t-1},s,y^{n}\right)f_{F_{t}}^{\left(\mu ,\lambda ,\theta \right)}\left(x_{t},y_{t},z_{t}|u_{t}\right).\\ \overset{\left(b\right)}{=}\sum_{u_{t},x_{t},y_{t},z_{t}}p_{U_{t}X_{t}Y_{t}Z_{t};F^{t-1}}^{\left(\mu ,\lambda ,\theta \right)}\left(u_{t},x_{t},y_{t},z_{t}\right)f_{F_{t}}^{\left(\mu ,\lambda ,\theta \right)}\left(x_{t},y_{t},z_{t}|u_{t}\right). \end{array}$$

Step (a) follows from Equation (33) in Lemma 5. Step (b) follows from the definition in Equation (37) of $p_{U_{t}X_{t}Y_{t}Z_{t};F^{t-1}}^{\left(\mu ,\lambda ,\theta \right)}$. ☐

The following proposition is a mathematical core to prove our main result.

**Proposition** **2.** *For θ∈[0,1), we choose the parameter α such that*

(43) $$\alpha =\frac{\theta }{1-\theta }\Leftrightarrow \theta =\frac{\alpha }{1+\alpha }.$$

*Then, for any λ≥0,μ∈[0,1] and for any θ∈[0,1), we have*

(44) $${\underline{\Omega }}^{\left(\mu ,\lambda ,\theta \right)}\left(p_{XY}\right)\geq \frac{\Omega^{\left(\mu ,\lambda ,\alpha \right)}\left(p_{XY}\right)}{1+\alpha }.$$

**Proof.**  Set

Q^n:={q=qUXYZ:|U|≤|Mn||Xn−1||Yn−1|},Ω^n(μ,λ,α)(pXY):=minq∈Q^nΩ(μ,λ,α)(q|pXY).

Then, by Lemma 6, we have

$$\begin{array}{ccc} \Lambda_{t,F^{t}}^{\left(\mu ,\lambda ,\theta \right)} & = & \sum_{u_{t},x_{t},y_{t},z_{t}}p_{U_{t}X_{t}Y_{t}Z_{t};F^{t-1}}^{\left(\mu ,\lambda ,\theta \right)}\left(u_{t},x_{t},y_{t},z_{t}\right)f_{F_{t}}^{\left(\mu ,\lambda ,\theta \right)}\left(x_{t},y_{t},z_{t}|u_{t}\right). \end{array}$$

For each t=1,2,⋯,n, we recursively choose $q_{t}=q_{U_{t}X_{t}Y_{t}Z_{t}}$ so that $q_{U_{t}X_{t}Y_{t}Z_{t}}=p_{U_{t}X_{t}Y_{t}Z_{t};F^{t-1}}^{\left(\mu ,\lambda ,\theta \right)}$ and choose $Q_{X_{t}}^{\left(i\right)}$, $Q_{Y_{t}|X_{t}U_{t}}^{\left(\mathrm{ii}\right)}$, $Q_{X_{t}|U_{t}Y_{t}Z_{t}}^{\left(\mathrm{iii}\right)}$, and $Q_{X_{t}|Y_{t}U_{t}}^{\left(\mathrm{iv}\right)}$ appearing in

$$\begin{array}{c} f_{F_{t}}^{\left(\mu ,\lambda ,\theta \right)}\left(x_{t},y_{t},z_{t}|u_{t}\right)\\ :={\left\{\frac{p_{X_{t}}\left(x_{t}\right)}{Q_{X_{t}}^{\left(i\right)}\left(x_{t}\right)}\frac{p_{Y_{t}|X_{t}}\left(y_{t}|x_{t}\right)}{Q_{Y_{t}|X_{t}U_{t}}^{\left(\mathrm{ii}\right)}\left(y_{t}|x_{t},u_{t}\right)}\frac{p_{X_{t}|U_{t}Y_{t}}\left(x_{t}|u_{t},y_{t}\right)}{Q_{X_{t}|U_{t}Y_{t}Z_{t}}^{\left(\mathrm{iii}\right)}\left(x_{t}|u_{t},y_{t},z_{t}\right)}{\left(\frac{p_{X_{t}|Y_{t}}\left(x_{t}|y_{t}\right)}{Q_{X_{t}|Y_{t}U_{t}}^{\left(\mathrm{iv}\right)}\left(x_{t}|u_{t},y_{t}\right)}\right)}^{\lambda \bar{\mu }}e^{-\lambda \mu d(x_{t},z_{t})}\right\}}^{\theta } \end{array}$$

such that they are the distributions induced by $q_{U_{t}X_{t}Y_{t}Z_{t}}$. Then, for each t=1,2,⋯, *n*, we have the following chain of inequalities:

(45) $$\begin{array}{c} \Lambda_{t,F^{t}}^{\left(\mu ,\lambda ,\theta \right)}=E_{q_{t}}\left[{\left\{\frac{p_{X_{t}}\left(X_{t}\right)}{q_{X_{t}}\left(X_{t}\right)}\frac{p_{Y_{t}|X_{t}}\left(Y_{t}|X_{t}\right)}{q_{Y_{t}|X_{t}U_{t}}\left(Y_{t}|X_{t},U_{t}\right)}\frac{p_{X_{t}|U_{t}Y_{t}}\left(X_{t}|U_{t},Y_{t}\right)}{q_{X_{t}|U_{t}Y_{t}Z_{t}}\left(X_{t}|U_{t},Y_{t},Z_{t}\right)}\frac{p_{X_{t}|Y_{t}}^{\lambda \bar{\mu }}\left(X_{t}|Y_{t}\right)e^{-\lambda \mu d(X_{t},Z_{t})}}{q_{X_{t}|Y_{t}U_{t}}^{\lambda \bar{\mu }}\left(X_{t}|U_{t},Y_{t}\right)}\right\}}^{\theta }\right]\\ =E_{q_{t}}\left[{\left\{\frac{p_{X_{t}}\left(X_{t}\right)}{q_{X_{t}}\left(X_{t}\right)}\frac{p_{Y_{t}|X_{t}}\left(Y_{t}|X_{t}\right)}{q_{Y_{t}|X_{t}U_{t}}\left(Y_{t}|X_{t},U_{t}\right)}\frac{q_{X_{t}|U_{t}Y_{t}}\left(X_{t}|U_{t},Y_{t}\right)}{q_{X_{t}|U_{t}Y_{t}Z_{t}}\left(X_{t}|U_{t},Y_{t},Z_{t}\right)}\frac{p_{X_{t}|Y_{t}}^{\lambda \bar{\mu }}\left(X_{t}|Y_{t}\right)e^{-\lambda \mu d(X_{t},Z_{t})}}{q_{X_{t}|Y_{t}U_{t}}^{\lambda \bar{\mu }}\left(X_{t}|U_{t},Y_{t}\right)}\right\}}^{\theta }\right.\\ \;\times \left.{\left\{\frac{p_{X_{t}|U_{t}Y_{t}}\left(X_{t}|U_{t},Y_{t}\right)}{q_{X_{t}|U_{t}Y_{t}}\left(X_{t}|U_{t},Y_{t}\right)}\right\}}^{\theta }\right]\\ \overset{\left(a\right)}{\leq }{\left(E_{q_{t}}\left[{\left\{\frac{p_{X_{t}}^{}\left(X_{t}\right)}{q_{X_{t}}^{}\left(X_{t}\right)}\frac{p_{Y_{t}|X_{t}}\left(Y_{t}|X_{t}\right)}{q_{Y_{t}|X_{t}U_{t}}\left(Y_{t}|X_{t},U_{t}\right)}\frac{q_{Z_{t}|U_{t}Y_{t}}\left(Z_{t}|U_{t},Y_{t}\right)}{q_{Z_{t}|U_{t}X_{t}Y_{t}}\left(Z_{t}|U_{t},X_{t},Y_{t}\right)}\frac{p_{X_{t}|Y_{t}}^{\lambda \bar{\mu }}\left(X_{t}|Y_{t}\right)e^{-\lambda \mu d(X_{t},Z_{t})}}{q_{X_{t}|Y_{t}U_{t}}^{\lambda \bar{\mu }}\left(X_{t}|U_{t},Y_{t}\right)}\right\}}^{\frac{\theta }{1-\theta }}\right]\right)}^{1-\theta }\\ \;\times {\left(E_{q_{t}}\left\{\frac{p_{X_{t}|U_{t}Y_{t}}\left(X_{t}|U_{t},Y_{t}\right)}{q_{X_{t}|U_{t}Y_{t}}\left(X_{t}|U_{t},Y_{t}\right)}\right\}\right)}^{\theta }=\exp\left\{-\left(1-\theta \right)\Omega^{\left(\mu ,\lambda ,\frac{\theta }{1-\theta }\right)}\left(q_{t}|p_{XY}\right)\right\}\\ \overset{\left(b\right)}{=}\exp\left\{-\frac{\Omega^{\left(\mu ,\lambda ,\alpha \right)}\left(q_{t}|p_{XY}\right)}{1+\alpha }\right\}\overset{\left(c\right)}{\leq }\exp\left\{-\frac{{\hat{\Omega }}_{n}^{\left(\mu ,\lambda ,\alpha \right)}\left(p_{XY}\right)}{1+\alpha }\right\}\overset{\left(d\right)}{=}\exp\left\{-\frac{\Omega^{\left(\mu ,\lambda ,\alpha \right)}\left(p_{XY}\right)}{1+\alpha }\right\}. \end{array}$$

Step (a) follows from Hölder’s inequality and the following identity:

$$\begin{array}{c} \frac{q_{X_{t}|U_{t}Y_{t}}\left(X_{t}|U_{t},Y_{t}\right)}{q_{X_{t}|U_{t}Y_{t}Z_{t}}\left(X_{t}|U_{t},Y_{t},Z_{t}\right)}=\frac{q_{Z_{t}|U_{t}Y_{t}}\left(Z_{t}|U_{t},Y_{t}\right)}{q_{Z_{t}|U_{t}X_{t}Y_{t}}\left(Z_{t}|U_{t},X_{t},Y_{t}\right)}\;\mathrm{for}\;t=1,2,\cdots ,n. \end{array}$$

Step (b) follows from Equation (43). Step (c) follows from the definition of ${\hat{\Omega }}_{n}^{\left(\mu ,\lambda ,\alpha \right)}$$\left(p_{XY}\right)$. Step (d) follows from that by Property 4 Part (a), the bound |U|≤|X|, is sufficient to describe ${\hat{\Omega }}_{n}^{\left(\mu ,\lambda ,\alpha \right)}\left(p_{XY}\right)$. Hence, we have the following:

(46) $$\begin{array}{c} \max_{q^{n}\in Q^{n}}\frac{1}{n}\Omega_{}^{\left(\mu ,\lambda ,\theta \right)}\left(p^{\left(n\right)},{\underline{Q}}^{n}|p_{XY}\right)\\ \geq \frac{1}{n}\Omega_{}^{\left(\mu ,\lambda ,\theta \right)}\left(p^{\left(n\right)},{\underline{Q}}^{n}|p_{XY}\right)\overset{\left(a\right)}{=}-\frac{1}{n}\sum_{t=1}^{n}\log\Lambda_{t,F^{t}}^{\left(\mu ,\lambda ,\theta \right)}\overset{\left(b\right)}{\geq }\frac{\Omega^{\left(\mu ,\lambda ,\alpha \right)}\left(p_{XY}\right)}{1+\alpha }.\; \end{array}$$

Step (a) follows from Equation (38) in Lemma 6. Step (b) follows from Equation (45). Since Equation (46) holds for any n≥1 and any $p^{\left(n\right)}\in P^{\left(n\right)}$$\left(p_{XY}\right)$, we have

$${\underline{\Omega }}^{\left(\mu ,\lambda ,\theta \right)}\left(p_{XY}\right)\geq \frac{\Omega^{\left(\mu ,\lambda ,\alpha \right)}\left(p_{XY}\right)}{1+\alpha }.$$

Thus, we have Equation (44) in Proposition 2. ☐

**Proof** **of** **Theorem** **3:** For θ∈[0,1), set

(47) $$\alpha =\frac{\theta }{1-\theta }\Leftrightarrow \theta =\frac{\alpha }{1+\alpha }.$$

Then, we have the following:

$$\begin{array}{cc} & \frac{1}{n}\log\left\{\frac{5}{P_{c}^{\left(n\right)}\left(\varphi^{\left(n\right)},\psi^{\left(n\right)};\Delta \right)}\right\}\overset{\left(a\right)}{\geq }\frac{{\underline{\Omega }}^{\left(\mu ,\lambda ,\theta \right)}\left(p_{XY}\right)-\lambda \theta \left(\bar{\mu }R+\mu \Delta \right)}{1+\theta (3+\lambda \bar{\mu })}\\ \overset{\left(b\right)}{\geq } & \frac{\frac{1}{1+\alpha }\Omega^{\left(\mu ,\lambda ,\alpha \right)}\left(p_{XY}\right)-\frac{\lambda \alpha }{1+\alpha }\left(\bar{\mu }R+\mu \Delta \right)}{1+\frac{\alpha }{1+\alpha }\left(3+\lambda \bar{\mu }\right)}=\frac{\Omega^{\left(\mu ,\lambda ,\alpha \right)}\left(p_{XY}\right)-\lambda \alpha \left(\bar{\mu }R+\mu \Delta \right)}{1+\alpha +\alpha (3+\lambda \bar{\mu })}\\ = & F^{\left(\mu ,\lambda ,\alpha \right)}\left(\bar{\mu }R+\mu \Delta |p_{XY}\right). \end{array}$$

Step (a) follows from Corollary 3. Step (b) follows from Proposition 2 and Equation (47). Since the above bound holds for any positive λ≥0, μ∈[0,1], and α≥0, we have

$$\frac{1}{n}\log\left\{\frac{5}{P_{c}^{\left(n\right)}\left(\varphi^{\left(n\right)},\psi^{\left(n\right)};\Delta \right)}\right\}\geq F\left(R,\Delta |p_{XY}\right).$$

Thus, Equation (10) in Theorem 3 is proved. ☐

**Proof** **of** **Corollary** **2:** Since *g* is an inverse function of ϑ, the definition in Equation (13) of $\kappa_{n}$ is equivalent to

(48) $$g\left(\frac{\kappa_{n}}{\rho (p_{XY})}\right)=\sqrt{\frac{10}{n\rho (p_{XY})}\log\left(\frac{5}{1-\varepsilon }\right)}.$$

By the definition of $n_{0}=n_{0}\left(\varepsilon ,\rho \left(p_{XY}\right)\right)$, we have that $\kappa_{n}\leq \left(1/2\right)\rho \left(p_{XY}\right)$ for $n\geq n_{0}$. We assume that for $n\geq n_{0}$, $\left(R,\Delta \right)\in R_{\mathrm{WZ}}\left(n,\varepsilon |p_{XY}\right).$ Then, there exists a sequence $\{(\varphi^{\left(n\right)},$$\psi^{\left(n\right)})$ ${\}}_{n\geq n_{0}}$ such that for $n\geq n_{0}$, we have

$$\frac{1}{n}\log||\varphi^{\left(n\right)}\left|\right|\leq R,\;P_{e}^{\left(n\right)}\left(\varphi^{\left(n\right)},\psi^{\left(n\right)};\Delta \right)\leq \varepsilon .$$

Then, by Theorem 3, we have

(49) $$\begin{array}{ccc} 1-\varepsilon & \leq & P_{c}^{\left(n\right)}\left(\varphi^{\left(n\right)},\psi^{\left(n\right)};\Delta \right)\leq 5\exp\left\{-nF(R,\Delta |p_{XY})\right\} \end{array}$$

for any $n\geq n_{0}$. We claim that for $n\geq n_{0}$, we have $\left(R+\kappa_{n},\Delta +\kappa_{n}\right)$ ∈ $R(p_{XY})$. To prove this claim, we suppose that $\left(R+\kappa_{n^{*}},\Delta +\kappa_{n^{*}}\right)$ does not belong to $R(p_{XY})$ for some $n^{*}\geq n_{0}$. Then, we have the following chain of inequalities:

(50) $$\begin{array}{cc} & 5\exp\left[-n^{*}F\left(R,\Delta |p_{XY}\right)\right]\overset{\left(a\right)}{<}5\exp\left[-\frac{n^{*}\rho \left(p_{XY}\right)}{10}\cdot g^{2}\left(\frac{\kappa_{n^{*}}}{\rho (p_{XY})}\right)\right]\\ \overset{\left(b\right)}{=} & 5\exp\left[-\frac{n^{*}\rho \left(p_{XY}\right)}{10}\frac{10}{n^{*}\rho \left(p_{XY}\right)}\log\left(\frac{5}{1-\varepsilon }\right)\right]=5\exp\left[\log\left(\frac{1-\varepsilon }{5}\right)\right]=1-\varepsilon . \end{array}$$

Step (a) follows from $\kappa_{n^{*}}\leq \left(1/2\right)\rho \left(p_{XY}\right)$ and Property 4 Part (e). Step (b) follows from Equation (48). The bound of Equation (50) contradicts Equation (49). Hence, we have $(R+\kappa_{n},\Delta +$$\kappa_{n})$ ∈ $R(p_{XY})$ or equivalent to

$$\left(R,\Delta \right)\in R\left(p_{XY}\right)-\kappa_{n}\left(1,1\right)$$

for $n\geq n_{0}$, which implies that for $n\geq n_{0}$,

$$R_{\mathrm{WZ}}\left(n,\varepsilon |p_{XY}\right)\subseteq R\left(p_{XY}\right)-\kappa_{n}\left(1,1\right),$$

completing the proof. ☐

## 5. Conclusions

For the WZ system, we have derived an explicit lower bound of the optimal exponent function on the correct probability of decoding for for $\left(R,\Delta \right)\notin R_{\mathrm{WZ}}\left(p_{XY}\right)$. We have described this result in Theorem 3. The determination problem of *the optimal* exponent remains to be resolved. This problem is our future work.

In this paper, we have treated the case where X and Y are finite sets. Extension of Theorem 3 to the case where X and Y are *arbitrary sets* is also our future work. Wyner [12] investigated the characterization of the rate distortion region in the case where X and Y are general sets and ${\left\{\left(X_{t},Y_{t}\right)\right\}}_{t=1}^{\infty }$ is a correlated stationary memoryless source. This work may provide a good tool to investigate the second future work.

## Figures and Tables

**Table 1:** Previous results on the converse coding theorems for DMS, WZ. Main results in the present paper on WZ are also included.

	Characterization of the Rate Distortion Region	Strong Converse	Strong Converse Exponent
DMS	Shannon [2] (1959) (Explicit form of ${\tilde{R}}_{\mathrm{DMS}}\left(p_{X}\right)$) Wolfowitz [3] (1966) (${\tilde{R}}_{\mathrm{DMS}}\left(p_{X}\right)=R_{\mathrm{DMS}}\left(p_{X}\right)$)	Wolfowitz [3] (1966) ($R_{\mathrm{DMS}}\left(\varepsilon |p_{X}\right)=R_{\mathrm{DMS}}\left(p_{X}\right)$ for any ε∈(0,1))	Csiszár and Körner [4] (1981) (The optimal exponent)
WZ	Wyner and Ziv [1] (1976) (Explicit form of ${\tilde{R}}_{\mathrm{WZ}}\left(p_{XY}\right)$) Csiszár and Körner [4] (1981) (${\tilde{R}}_{\mathrm{WZ}}\left(p_{XY}\right)=R_{\mathrm{WZ}}\left(p_{XY}\right)$)	Corollary 2 (Outer bound with $O\left(1/\sqrt{n}\right)$ gap from the rate distortion region, $R_{\mathrm{WZ}}\left(\varepsilon |p_{XY}\right)=R_{\mathrm{WZ}}\left(p_{XY}\right)$ for any ε∈(0,1))	Theorem 3 (Lower bound *F* of the opt. exp. *G*)

## Appendix A. Properties of the Rate Distortion Regions

In this Appendix, we prove Property 1. Property 1 Part (a) can easily be proved by the definitions of the rate distortion regions. We omit the proofs of this part. In the following argument, we prove Part (b).

**Proof** **of** **Property** **1** **Part** **(b):** We set

$$\begin{array}{ccc} {\underline{R}}_{\mathrm{WZ}}\left(m,\varepsilon |p_{XY}\right) & = & \underset{n\geq m}{⋂}R_{\mathrm{WZ}}\left(n,\varepsilon |p_{XY}\right). \end{array}$$

By the definitions of ${\underline{R}}_{\mathrm{WZ}}\left(m,\varepsilon |p_{XY}\right)$ and $R_{\mathrm{WZ}}\left(\varepsilon |p_{XY}\right)$, we have that ${\underline{R}}_{\mathrm{WZ}}\left(m,\varepsilon |p_{XY}\right)$ $\subseteq R_{\mathrm{WZ}}\left(\varepsilon |p_{XY}\right)$ for m≥1. Hence, we have that

(A1) $$\underset{m\geq 1}{⋃}{\underline{R}}_{\mathrm{WZ}}\left(m,\varepsilon |p_{XY}\right)\subseteq R_{\mathrm{WZ}}\left(\varepsilon |p_{XY}\right).$$

We next assume that $\left(R,\Delta \right)\in R_{\mathrm{WZ}}\left(\varepsilon |p_{XY}\right)$. Set

$$R_{\mathrm{WZ}}^{\left(\delta \right)}\left(\varepsilon |p_{XY}\right):=\left\{\left(R+\delta ,\Delta \right):\left(R,\Delta \right)\in R_{\mathrm{WZ}}\left(\varepsilon |p_{XY}\right)\right\}.$$

Then, by the definitions of $R_{\mathrm{WZ}}(n,\varepsilon$$|p_{XY})$ and $R_{\mathrm{WZ}}($$\varepsilon |p_{XY})$, we have that, for any δ>0, there exists $n_{0}\left(\varepsilon ,\delta \right)$ such that for any $n\geq n_{0}\left(\varepsilon ,\delta \right)$, $\left(R+\delta ,\Delta \right)\in R_{\mathrm{WZ}}\left(n,\varepsilon |p_{XY}\right),$ which implies that

(A2) $$\begin{array}{cc} & R_{\mathrm{WZ}}^{\left(\delta \right)}\left(\varepsilon |p_{XY}\right)\subseteq \underset{n\geq n_{0}\left(\varepsilon ,\delta \right)}{⋂}R_{\mathrm{WZ}}\left(n,\varepsilon |p_{XY}\right)\\ = & {\underline{R}}_{\mathrm{WZ}}\left(n_{0}\left(\varepsilon ,\delta \right),\varepsilon |p_{XY}\right)\subseteq \mathrm{cl}\left(\underset{m\geq 1}{⋃}{\underline{R}}_{\mathrm{WZ}}\left(m,\varepsilon |p_{XY}\right)\right). \end{array}$$

Here, we assume that there exists a pair (R,Δ) belonging to $R_{\mathrm{WZ}}\left(\varepsilon |p_{XY}\right)$ such that

(A3) $$\left(R,\Delta \right)\notin \mathrm{cl}\left(\underset{m\geq 1}{⋃}{\underline{R}}_{\mathrm{WZ}}\left(m,\varepsilon |p_{XY}\right)\right).$$

Since the set in the right hand side of Equation (A3) is a closed set, we have

(A4) $$\left(R+\delta ,\Delta \right)\notin \mathrm{cl}\left(\underset{m\geq 1}{⋃}{\underline{R}}_{\mathrm{WZ}}\left(m,\varepsilon |p_{XY}\right)\right)$$

for some small δ>0. Note that (R+δ,Δ) $\in R_{\mathrm{WZ}}^{\left(\delta \right)}\left(\varepsilon |p_{XY}\right)$. Then, Equation (A4) contradicts Equation (A2). Thus, we have

(A5) $$\begin{array}{ccc} \underset{m\geq 1}{⋃}{\underline{R}}_{\mathrm{WZ}}\left(m,\varepsilon |p_{XY}\right) & \subseteq & R_{\mathrm{WZ}}\left(\varepsilon |p_{XY}\right)\subseteq \mathrm{cl}\left(\underset{m\geq 1}{⋃}{\underline{R}}_{\mathrm{WZ}}\left(m,\varepsilon |p_{XY}\right)\right).\; \end{array}$$

Note here that $R_{\mathrm{WZ}}\left(\varepsilon |p_{XY}\right)$ is a closed set. Then, from Equation (A5), we conclude that

$$\begin{array}{ccc} R_{\mathrm{WZ}}\left(\varepsilon |p_{XY}\right) & = & \mathrm{cl}\left(\underset{m\geq 1}{⋃}{\underline{R}}_{\mathrm{WZ}}\left(m,\varepsilon |p_{XY}\right)\right)=\mathrm{cl}\left(\underset{m\geq 1}{⋃}\underset{n\geq m}{⋂}R_{\mathrm{WZ}}\left(n,\varepsilon |p_{XY}\right)\right), \end{array}$$

completing the proof. ☐

## Appendix B. Cardinality Bound on Auxiliary Random Variables

Set

$$\begin{array}{c} R^{\left(\mu \right)}\left(p_{XY}\right):=\min_{q\in P_{\mathrm{sh}}\left(p_{XY}\right)}\left\{\bar{\mu }I_{q}\left(X;U|Y\right)+\mu E_{q}d\left(X,Z\right)\right\},\\ {\underline{R}}^{\left(\mu \right)}\left(p_{XY}\right):=\min_{q\in P(p_{XY})}\left\{\bar{\mu }I_{q}\left(X;U|Y\right)+\mu E_{q}d\left(X,Z\right)\right\}, \end{array}$$

Since $P_{\mathrm{sh}}\left(p_{XY}\right)$ $\subseteq P(p_{XY})$, it is obvious that

(A6) $$R^{\left(\mu \right)}\left(p_{XY}\right)\geq {\underline{R}}^{\left(\mu \right)}\left(p_{XY}\right).$$

We first prove the following lemma.

**Lemma** **A1.**

$$\begin{array}{c} R^{\left(\mu \right)}\left(p_{XY}\right)={\underline{R}}^{\left(\mu \right)}\left(p_{XY}\right). \end{array}$$

To prove Lemma A1, we set

$$\begin{array}{c} P_{1}\left(p_{XY}\right):=\{\begin{array}{c} q_{1}=q_{UXY}:|U|\leq |X|,q_{XY}=p_{XY},U↔X↔Y\}, \end{array}\\ Q_{1}\left(p_{XY}\right):=\{\begin{array}{c} q_{1}=q_{UXY}:|U|\leq \left|X\right|+1,q_{XY}=p_{XY},U↔X↔Y\}, \end{array}\\ Q_{2}\left(q_{UXY}\right):=\left\{q_{2}=q_{Z|UXY}:q_{UXYZ}=\left(q_{UXY},q_{2}\right),X↔\left(U,Y\right)↔Z\right\}. \end{array}$$

By definition, it is obvious that

(A7) $$\begin{array}{ccc} P_{\mathrm{sh}}\left(p_{XY}\right) & = & \{q=\left(q_{1},q_{2}\right):q_{1}\in P_{1}\left(p_{XY}\right),q_{2}\in Q_{2}\left(q_{1}\right)\}, \end{array}$$

(A8) $$\begin{array}{ccc} P(p_{XY}) & = & \{q=\left(q_{1},q_{2}\right):q_{1}\in Q_{1}\left(p_{XY}\right),q_{2}\in Q_{2}\left(q_{1}\right)\}. \end{array}$$

**Proof** **of** **Lemma** **A1:** Since we have Equation (A6), it suffices to show $R^{\left(\mu \right)}\left(p_{XY}\right)\leq {\underline{R}}^{\left(\mu \right)}\left(p_{XY}\right)$ to prove Lemma A1. We bound the cardinality |U| of *U* to show that the bound |U|≤|X| is sufficient to describe ${\underline{R}}^{\left(\mu \right)}\left(p_{XY}\right)$. We first observe that, by Equation (A8), we have

$$\begin{array}{ccc} {\underline{R}}^{\left(\mu \right)}\left(p_{XY}\right) & = & \min_{q_{1}\in Q_{1}\left(p_{XY}\right)}\min_{q_{2}\in Q_{2}\left(q_{1}\right)}\left\{\bar{\mu }I_{q_{1}}\left(X;U|Y\right)\right.\left.+\mu E_{\left(q_{1},q_{2}\right)}d\left(X,Z\right)\right\}\\ & = & \min_{q_{1}\in Q_{1}\left(p_{XY}\right)}\{\bar{\mu }I_{q_{1}}\left(X;U|Y\right)\left.+\mu \min_{q_{2}\in Q_{2}\left(q_{1}\right)}E_{\left(q_{1},q_{2}\right)}d\left(X,Z\right)\right\}\\ & = & \min_{q_{1}\in Q_{1}\left(p_{XY}\right)}\left\{\bar{\mu }I_{q_{1}}\left(X;U|Y\right)+\mu E_{\left(q_{1},q_{2}^{*}\left(q_{1}\right)\right)}d\left(X,Z\right)\right\}, \end{array}$$

where

$$q_{2}^{*}=q_{2}^{*}\left(q_{1}\right)=q_{Z|UY}^{*}={\left\{q_{Z|UY}^{*}\left(z|u,y\right)\right\}}_{(u,y,z)\in U\times Y\times Z}$$

is a conditional probability distribution that attains the following optimization problem:

$$\min_{q_{2}\in Q_{2}\left(q_{1}\right)}E_{\left(q_{1},q_{2}\right)}d\left(X,Z\right).$$

Observe that

(A9) $$\begin{array}{c} p_{X}\left(x\right)=\sum_{u\in U}q_{U}\left(u\right)q_{X|U}\left(x|u\right), \end{array}$$

(A10) $$\begin{array}{c} \bar{\mu }I_{q_{1}}\left(X;U|Y\right)+\mu E_{\left(q_{1},q_{2}^{*}\right)}\left(X,Z\right)=\sum_{u\in U}p_{U}\left(u\right)\pi \left(q_{X|U}\left(\cdot |u\right)\right), \end{array}$$

where

$$\begin{array}{ccc} \pi (q_{X|U}\left(\cdot |u\right)) & := & \sum_{(x,y,z)\in X\times Y\times Z}q_{X|U}\left(x|u\right)p_{Y|X}\left(y|x\right)q_{Z|UY}^{*}\left(z|u,y\right)\\ & & \times \log\left\{\frac{q_{X|U}^{\bar{\mu }}\left(x|u\right)}{p_{X}^{\bar{\mu }}\left(x\right)}\frac{p_{Y}^{\bar{\mu }}\left(y\right)e^{-\mu d(x,z)}}{{\left[\sum_{\tilde{x}\in X}p_{Y|X}\left(y|\tilde{x}\right)q_{X|U}\left(\tilde{x}|u\right)\right]}^{\bar{\mu }}}\right\}. \end{array}$$

For each u∈U, $\pi (q_{X|U}\left(\cdot |u\right))$ is a continuous function of $q_{X|U}\left(\cdot |u\right)$. Then, by the support lemma,

|U|≤|X|−1+1=|X|

is sufficient to express |X|−1 values of Equation (A9) and one value of Equation (A10). ☐

Next, we give a proof of Property 4 Part (a).

**Proof** **of** **Property** **4** **Part** **(a):** We first bound the cardinality |U| of *U* in Q to show that the bound |U|≤|X| is sufficient to describe $\Omega^{\left(\mu ,\lambda ,\alpha \right)}$$\left(p_{XY}\right)$. Observe that

(A11) $$\begin{array}{c} q_{X}\left(x\right)=\sum_{u\in U}q_{U}\left(u\right)q_{X|U}\left(x|u\right), \end{array}$$

(A12) $$\begin{array}{c} \exp\left\{-\Omega^{\left(\mu ,\lambda ,\alpha \right)}\left(q|p_{XY}\right)\right\}=\sum_{u\in U}q_{U}\left(u\right)\Pi^{\left(\mu ,\lambda ,\alpha \right)}\left(q_{X},q_{XYZ|U}\left(\cdot ,\cdot ,\cdot |u\right)\right), \end{array}$$

where

Π(μ,λ,α)(qX,qXYZ|U(·,·,·|u)):=∑(x,y,z)∈X×Y×ZqXYZ|U(x,y,z|u)exp−αωq||p(μ,λ)(x,y,z|u).

The value of $q_{X}$ included in $\Pi^{\left(\mu ,\lambda ,\alpha \right)}\left(q_{X},q_{XYZ|U}\left(\cdot ,\cdot ,\cdot |u\right)\right)$ must be preserved under the reduction of U. For each u∈U, $\Pi^{\left(\mu ,\lambda \right)}\left(q_{X},q_{XYZ|U}\left(\cdot ,\cdot ,\cdot |u\right)\right)$ is a continuous function of $q_{XYZ|U}\left(\cdot ,\cdot ,\cdot |u\right)$. Then, by the support lemma,

|U|≤|X|−1+1=|X|

is sufficient to express |X|−1 values of Equation (A11) and one value of Equation (A12). We next bound the cardinality |U| of *U* in $P_{\mathrm{sh}}\left(p_{XY}\right)$ to show that the bound |U|≤|X| is sufficient to describe ${\tilde{\Omega }}^{\left(\mu ,\lambda \right)}$ $\left(p_{XY}\right)$. Observe that

(A13) $$\begin{array}{c} p_{X}\left(x\right)=\sum_{u\in U}p_{U}\left(u\right)p_{X|U}\left(x|u\right), \end{array}$$

(A14) $$\begin{array}{c} \exp\left\{-{\tilde{\Omega }}^{\left(\mu ,\lambda \right)}\left(p\right)\right\}=\sum_{u\in U}p_{U}\left(u\right){\tilde{\Pi }}^{\left(\mu ,\lambda \right)}\left(p_{X},p_{XYZ|U}\left(\cdot ,\cdot ,\cdot |u\right)\right), \end{array}$$

where

Π˜(μ,λ)(pX,pXYZ|U(·,·,·|u)):=∑(x,y,z)∈X×Y×ZpXYZ|U(x,y,z|u)exp−λωp(μ)(x,y,z|u).

The value of $p_{X}$ included in ${\tilde{\Pi }}^{\left(\mu ,\lambda \right)}\left(p_{X},p_{XYZ|U}\left(\cdot ,\cdot ,\cdot |u\right)\right)$ must be preserved under the reduction of U. For each u∈U, $\Pi^{\left(\mu ,\lambda \right)}\left(p_{X},p_{XYZ|U}\left(\cdot ,\cdot ,\cdot |u\right)\right)$ is a continuous function of $p_{XYZ|U}\left(\cdot ,\cdot ,\cdot |u\right)$. Then, by the support lemma,

|U|≤|X|−1+1=|X|

is sufficient to express |X|−1 values of Equation (A13) and one value of Equation (A14). ☐

## Appendix C. Proof of Property 2

In this Appendix, we prove Property 2. Property 2 Part (a) is a well known property. Proof of this property is omitted here. We only prove Property 2 Part (b).

**Proof** **of** **Property** **2** **Part** **(b):** Since $P^{*}\left(p_{XY}\right)$⊆$P(p_{XY})$, it is obvious that $R^{*}\left(p_{XY}\right)$⊆$R(p_{XY})$. Hence it suffices to prove that $R(p_{XY})$⊆$R^{*}\left(p_{XY}\right).$ We assume that $\left(R,\Delta \right)\in R\left(p_{XY}\right)$. Then, there exists $p\in P(p_{XY})$ such that

(A15) $$R\geq I_{p}\left(U;X|Y\right)and\Delta \geq E_{p}d\left(X,Z\right).$$

On the second inequality in Equation (A15), we have the following:

(A16) $$\begin{array}{ccc} \Delta & \geq & E_{p}d\left(X,Z\right)=\sum_{(u,y)\in U\times Y}p_{UY}\left(u,y\right)\left[\sum_{z\in Z}p_{Z|UY}\left(z|u,y\right)\right.\left.\left(\sum_{x\in X}d\left(x,z\right)p_{X|UY}\left(x|u,y\right)\right)\right]\\ & \geq & \sum_{(u,y)\in U\times Y}p_{UY}\left(u,y\right)\left[\min_{z\in Z}\left(\sum_{x\in X}d\left(x,z\right)p_{X|UY}\left(x|u,y\right)\right)\right]\\ & = & \sum_{(u,y)\in U\times Y}p_{UY}\left(u,y\right)\left(\sum_{x\in X}d\left(x,z^{*}\right)p_{X|UY}\left(x|u,y\right)\right),\; \end{array}$$

where $z^{*}=z^{*}\left(u,y\right)$ is one of the minimizers of the function

$$\sum_{x\in X}d\left(x,z\right)p_{X|UY}\left(x|u,y\right).$$

Define ϕ:U×Y→Z by $\phi \left(u,y\right)=z^{*}$. We further define $q=q_{UXYZ}$ by $q_{UXY}=p_{UXY},q_{Z}=q_{\phi (U,Y)}.$ It is obvious that

(A17) $$q\in P^{*}\left(p_{XY}\right)\mathrm{and}R\geq I_{p}\left(X;U|Y\right)=I_{q}\left(X;U|Y\right).$$

Furthermore, from Equation (A16), we have

(A18) $$\Delta \geq E_{p}d\left(X,Z\right)\geq E_{q}d\left(X,\phi \left(U,Y\right)\right).$$

From Equations (A17) and (A18), we have $\left(R,\Delta \right)\in R^{*}\left(p_{XY}\right)$. Thus $R(p_{XY})$⊆ $R^{*}\left(p_{XY}\right)$ is proved.

## Appendix D. Proof of Property 3

In this Appendix, we prove Property 3. From Property 2 Part (a), we have the following lemma.

**Lemma** **A2.**
*Suppose that $\left(\hat{R},\hat{\Delta }\right)$ does not belong to R($p_{XY})$. Then, there exist ϵ>0 and $\mu^{*}\in \left[0,1\right]$ such that for any $\left(R,\Delta \right)\in R\left(p_{XY}\right)$ we have*

$$\begin{array}{c} \bar{\mu^{*}}\left(R-\hat{R}\right)+\mu^{*}\left(\Delta -\hat{\Delta }\right)-\epsilon \geq 0. \end{array}$$

Proof of this lemma is omitted here. Lemma A2 is equivalent to the fact that if the region $R(p_{XY})$ is a convex set, then for any point $\left(\hat{R},\hat{\Delta }\right)$ outside the region $R(p_{XY})$, there exits a line which separates the point $\left(\hat{R},\hat{\Delta }\right)$ from the region $R(p_{XY})$. Lemma A2 will be used to prove Equation (8) in Property 3.

**Proof** **of** **Equation** **(8)** **in** **Property** **3:** We first recall the following definitions of $P(p_{XY})$ and $P_{\mathrm{sh}}\left(p_{XY}\right)$:

$$\begin{array}{ccc} P(p_{XY}) & := & \{p_{UXYZ}:|U|\leq |X|+1,U↔X↔Y,X↔\left(U,Y\right)↔Z\},\\ P_{\mathrm{sh}}\left(p_{XY}\right) & := & \{p_{UXYZ}:|U|\leq |X|,U↔X↔Y,X↔\left(U,Y\right)↔Z\}. \end{array}$$

We prove $R_{\mathrm{sh}}\left(p_{XY}\right)$$\subseteq R(p_{XY})$. We assume that $\left(\hat{R},\hat{\Delta }\right)\notin R\left(p_{XY}\right)$. Then, by Lemma A2, there exist ϵ>0 and $\mu^{*}\in \left[0,1\right]$ such that for any $\left(R,\Delta \right)\in R\left(p_{XY}\right)$, we have

$$\begin{array}{c} {\bar{\mu }}^{*}\hat{R}+\mu^{*}\hat{\Delta }\leq {\bar{\mu }}^{*}R+\mu^{*}\Delta -\epsilon . \end{array}$$

Hence, we have

(A19) $$\begin{array}{cc} & {\bar{\mu }}^{*}\hat{R}+\mu^{*}\hat{\Delta }\leq \min_{\left(R,\Delta \right)\in R\left(p_{XY}\right)}\left\{{\bar{\mu }}^{*}R+\mu^{*}\Delta \right\}-\epsilon \\ \overset{\left(a\right)}{=} & \min_{p\in P(p_{XY})}\left\{{\bar{\mu }}^{*}I_{p}\left(U;X|Y\right)+\mu^{*}E_{p}d\left(X,Z\right)\right\}-\epsilon \\ \leq & \min_{p\in P_{\mathrm{sh}}\left(p_{XY}\right)}\left\{{\bar{\mu }}^{*}I_{p}\left(U;X|Y\right)+\mu^{*}E_{p}d\left(X,Z\right)\right\}-\epsilon =R^{\left(\mu^{*}\right)}\left(p_{XY}\right)-\epsilon . \end{array}$$

Step (a) follows from the definition of $R(p_{XY})$. The inequality in Equation (A19) implies that $\left(\hat{R},\hat{\Delta }\right)$$\notin R_{\mathrm{sh}}\left(p_{XY}\right)$. Thus, $R_{\mathrm{sh}}\left(p_{XY}\right)$$\subseteq R(p_{XY})$ is concluded. We next prove $R\left(p_{XY}\right)$ ⊆ $R_{\mathrm{sh}}$$\left(p_{XY}\right)$. We assume that $\left(R,\Delta )\in R(p_{XY}\right)$. Then, there exists $q\in P\left(p_{XY}\right)$ such that

(A20) $$R\geq I_{q}\left(X;U|Y\right),\Delta \geq E_{q}d\left(X,Z\right).$$

Then, for each μ∈[0,1] and for (R,Δ)$\in R(p_{XY})$, we have the following chain of inequalities:

$$\begin{array}{cc} & \bar{\mu }R+\mu \Delta \overset{\left(a\right)}{\geq }\bar{\mu }I_{q}\left(X;U|Y\right)+\mu E_{q}d\left(X,Z\right)\\ \geq & \min_{q\in P(p_{XY})}\left[\bar{\mu }I_{q}\left(X;U|Y\right)+\mu E_{q}d\left(X,Z\right)\right]={\underline{R}}^{\left(\mu \right)}\left(p_{XY}\right)\overset{\left(b\right)}{=}R^{\left(\mu \right)}\left(p_{XY}\right). \end{array}$$

Step (a) follows from Equation (A20). Step (b) follows from Lemma A1. Hence, we have $R(p_{XY})$⊆ $R_{\mathrm{sh}}\left(p_{XY}\right)$. ☐

## Appendix E. Proof of Property 4 Part (b)

In this Appendix, we prove Property 4 Part (b). We have the following lemma.

**Lemma** **A3.**
*For any μ∈[0,1], λ≥0, $\alpha \in [0,\frac{1}{\lambda +1}]$ and any $q=q_{UXY}\in Q\left(p_{Y|X}\right)$, there exists $p=p_{UXYZ}\in P_{\mathrm{sh}}\left(p_{XY}\right)$ such that*

(A21) $$\Omega^{\left(\mu ,\gamma ,\alpha \right)}\left(q|p_{XY}\right)\geq \alpha {\tilde{\Omega }}^{\left(\mu ,\lambda \right)}\left(p\right).$$

*This implies that for any μ∈[0,1], λ≥0, $\alpha \in [0,\frac{1}{\lambda +1}]$, we have*

(A22) $$\Omega^{\left(\mu ,\gamma ,\alpha \right)}\left(p_{XY}\right)\geq \alpha {\tilde{\Omega }}^{\left(\mu ,\lambda \right)}\left(p_{XY}\right).$$

**Proof.** Since Equation (A22) is obvious from Equation (A21), we only prove Equation (A21). We consider the case where (μ,λ,α) satisfies (μ,λ)∈[0,1], λ≥0, and $\alpha \in [0,\frac{1}{1+\lambda }]$. In this case, we have

(A23) $$\lambda \bar{\mu }\frac{\alpha }{\bar{\alpha }}\leq \frac{\lambda \alpha }{\bar{\alpha }}\leq \frac{\frac{\lambda }{1+\lambda }}{1-\frac{1}{1+\lambda }}=1.$$

For each $q=q_{UXYZ}\in Q$, we choose $p=p_{UXYZ}\in P_{\mathrm{sh}}\left(p_{XY}\right)$ so that $p_{U|X}=q_{U|X}$ and $p_{Z|UY}=q_{Z|UY}$. Then, for any (μ,λ,α) satisfying μ∈[0,1], λ≥0, and $\alpha \in [0,\frac{1}{1+\lambda }]$, we have the following chain of inequalities:

(A24) $$\begin{array}{c} \exp\left\{-\Omega^{\left(\mu ,\lambda ,\alpha \right)}\left(q|p_{XY}\right)\right\}\\ =E_{q}\left[{\left\{\frac{p_{X}\left(X\right)}{q_{X}\left(X\right)}\frac{p_{Y|X}\left(Y|X\right)}{q_{Y|XU}\left(Y|X,U\right)}\frac{q_{Z|UY}\left(Z|U,Y\right)}{q_{Z|UYX}\left(Z|U,Y,X\right)}\frac{p_{X|Y}^{\lambda \bar{\mu }}\left(X|Y\right)e^{-\lambda \mu d(X,Z)}}{q_{X|UY}^{\lambda \bar{\mu }}\left(X|U,Y\right)}\right\}}^{\alpha }\right]\\ =E_{q}\left[{\left\{\frac{p_{UXYZ}\left(X,Y,Z,U\right)}{q_{UXYZ}\left(X,Y,Z,U\right)}\frac{p_{X|Y}^{\lambda \bar{\mu }}\left(X|Y\right)e^{-\lambda \mu d(X,Z)}}{p_{X|UY}^{\lambda \bar{\mu }}\left(X|U,Y\right)}\right\}}^{\alpha }{\left\{\frac{p_{X|UY}\left(X|U,Y\right)}{q_{X|UY}\left(X|U,Y\right)}\right\}}^{\lambda \bar{\mu }\alpha }\right]\\ \overset{\left(a\right)}{\leq }{\left(E_{q}\left[\frac{p_{UXYZ}\left(X,Y,Z,U\right)}{q_{UXYZ}\left(X,Y,Z,U\right)}\frac{p_{X|Y}^{\lambda \bar{\mu }}\left(X|Y\right)e^{-\lambda \mu d(X,Z)}}{p_{X|UY}^{\lambda \bar{\mu }}\left(X|U,Y\right)}\right]\right)}^{\alpha }{\left(E_{q}\left[{\left\{\frac{p_{X|UY}\left(X|U,Y\right)}{q_{X|UY}\left(X|U,Y\right)}\right\}}^{\lambda \bar{\mu }\frac{\alpha }{\bar{\alpha }}}\right]\right)}^{\bar{\alpha }}\\ =\exp\left\{-\alpha {\tilde{\Omega }}^{\left(\mu ,\lambda \right)}\left(p\right)\right\}{\left(E_{q}\left[{\left\{\frac{p_{X|UY}\left(X|U,Y\right)}{q_{X|UY}\left(X|U,Y\right)}\right\}}^{\lambda \bar{\mu }\frac{\alpha }{\bar{\alpha }}}\right]\right)}^{\bar{\alpha }}\overset{\left(b\right)}{\leq }\exp\left\{-\alpha {\tilde{\Omega }}^{\left(\mu ,\lambda \right)}\left(p\right)\right\}. \end{array}$$

Step (a) follows from Hölder’s inequality. Step (b) follows from Equation (A23) and Hölder’s inequality. ☐

**Proof** **of** **Property** **4** **Part** **(b):** We have the following chain of inequalities:

$$\begin{array}{c} F\left(R,\Delta |p_{XY}\right)=\underset{\mu \in [0,1],\lambda ,\alpha \geq 0}{\sup}\frac{\Omega^{\left(\mu ,\lambda ,\alpha \right)}\left(p_{XY}\right)-\lambda \alpha \left(\bar{\mu }R+\mu \Delta \right)}{1+(4+\bar{\mu }\lambda )\alpha }\\ \geq \underset{\mu \in [0,1],\lambda \geq 0}{\sup}\underset{\alpha \in \left[0,\frac{1}{1+\lambda }\right]}{\sup}\frac{\Omega^{\left(\mu ,\lambda ,\alpha \right)}\left(p_{XY}\right)-\lambda \alpha \left(\bar{\mu }R+\mu \Delta \right)}{1+(4+\bar{\mu }\lambda )\alpha }\\ \overset{\left(a\right)}{\geq }\underset{\mu \in [0,1],\lambda \geq 0}{\sup}\underset{\alpha \in \left[0,\frac{1}{1+\lambda }\right]}{\sup}\frac{\alpha [{\tilde{\Omega }}^{\left(\mu ,\lambda \right)}\left(p_{XY}\right)-\lambda \left(\bar{\mu }R+\mu \Delta \right)]}{1+(4+\bar{\mu }\lambda )\alpha }\\ \overset{\left(b\right)}{=}\underset{\mu \in [0,1],\lambda \geq 0}{\sup}\frac{{\tilde{\Omega }}^{\left(\mu ,\lambda \right)}\left(p_{XY}\right)-\lambda \left(\bar{\mu }R+\mu \Delta \right)}{5+\lambda (1+\bar{\mu })}=\tilde{F}\left(R,\Delta |p_{XY}\right). \end{array}$$

Step (a) follows from Lemma A3. Step (b) follows from

$$\underset{\alpha \in \left[0,\frac{1}{1+\lambda }\right]}{\sup}\frac{\alpha }{1+(4+\bar{\mu }\lambda )\alpha }=\frac{1}{5+\lambda (1+\bar{\mu })},$$

completing the proof. ☐

## Appendix F. Proof of Property 4 Parts (c), (d), and (e)

In this Appendix, we prove Property 4 Parts (c), (d), and (e). We first prove Parts (c) and (d).

**Proof** **of** **Property** **4** **Parts** **(c)** **and** **(d):** We first prove Part (c). We first show that for each $p\in P_{\mathrm{sh}}\left(p_{XY}\right)$ and μ∈[0,1], ${\tilde{\Omega }}^{\left(\mu ,\lambda \right)}\left(p\right)$ exists for λ∈[0,1]. On a lower bound of $\exp[-{\tilde{\Omega }}^{\left(\mu ,\lambda \right)}\left(p\right)]$, for λ∈[0,1], we have the following:

(A25) exp[−Ω˜(μ,λ)(p)]=∑(u,x,y,z)∈U×X×Y×ZpUXYZ(u,x,y,z)pX|Y(x|y)pX|UY(x|u,y)μ¯λe−μλd(x,z)

(A26) ≥(a)∑(u,x,y,z)∈U×X×Y×YpUXYZ(u,x,y,z)pX|Y(x|y)e−d(x,z).

Step (a) follows from that, for μ,λ∈[0,1],

$${\left[\frac{p_{X|Y}\left(x|y\right)}{p_{X|UY}\left(x|u,y\right)}\right]}^{\bar{\mu }\lambda }e^{-\mu \lambda d(x,z)}\geq p_{X|Y}\left(x|y\right)e^{-d(x,z)}.$$

It is obvious that the lower bound of Equation (A26) of $\exp[-{\tilde{\Omega }}^{\left(\mu ,\lambda \right)}\left(p\right)]$ takes some positive value. This implies that ${\tilde{\Omega }}^{\left(\mu ,\lambda \right)}\left(p\right)$ exists for λ∈[0,1]. We next show that ${\tilde{\Omega }}^{\left(\mu ,\lambda \right)}\left(p\right)\geq 0$ for λ∈[0,1]. On upper bounds of $\exp[-{\tilde{\Omega }}^{\left(\mu ,\lambda \right)}\left(p\right)]$ for λ∈[0,1], we have the following chain of inequalities:

(A27) exp[−Ω˜(μ,λ)(p)]≤(a)∑(u,x,y,z)∈U×X×Y×ZpUXYZ(u,x,y,z)pX|Y(x|y)pX|UY(x|u,y)μ¯λ=∑(u,x,y)∈U×X×YpUY(u,y)pX|UY1−μ¯λ(x|u,y)pX|Yμ¯λ(x|y)≤(b)∑(u,y)∈U×YpUY(u,y)∑x∈XpX|UY(x|u,y)1−μ¯λ∑x∈XpX|Y(x|y)μ¯λ=∑(u,y)∈U×YpUY(u,y)=1.

Step (a) follows from Equation (A25) and $e^{-\mu \lambda d(x,z)}\leq 1$. Step (b) follows from $\bar{\mu }\lambda \in \left[0,1\right]$ and Hölder’s inequality. We next prove that that, for each $p\in P_{\mathrm{sh}}\left(p_{XY}\right)$ and μ∈[0,1], ${\tilde{\Omega }}^{\left(\mu ,\lambda \right)}\left(p\right)$ is twice differentiable for λ∈[0,1/2]. For simplicity of notations, set

$$\begin{array}{c} \underline{a}:=\left(u,x,y,z\right),\underline{A}:=\left(U,X,Y,Z\right),\underline{A}:=U\times X\times Y\times Z,\\ {\tilde{\omega }}_{p}^{\left(\mu \right)}\left(x,y,z|u\right):=\varsigma \left(\underline{a}\right),{\tilde{\Omega }}^{\left(\mu ,\lambda \right)}\left(p\right):=\xi \left(\lambda \right). \end{array}$$

Then, we have

(A28) $${\tilde{\Omega }}^{\left(\mu ,\lambda \right)}\left(p\right)=\xi \left(\lambda \right)=-\log\left[\sum_{\underline{a}\in \underline{A}}p_{\underline{A}}\left(\underline{a}\right)e^{-\lambda \varsigma (\underline{a})}\right].$$

The quantity $p^{\left(\lambda \right)}\left(\underline{a}\right)=p_{\underline{A}}^{\left(\lambda \right)}\left(\underline{a}\right),\underline{a}\in A$ has the following form:

(A29) $$p^{\left(\lambda \right)}\left(\underline{a}\right)=e^{\xi (\lambda )}p\left(\underline{a}\right)e^{-\lambda \varsigma (\underline{a})}.$$

By simple computations, we have

(A30) $$\begin{array}{c} \xi^{\prime }\left(\lambda \right)=e^{\xi (\lambda )}\left[\sum_{\underline{a}\in \underline{A}}p\left(\underline{a}\right)\varsigma \left(\underline{a}\right)e^{-\lambda \varsigma (\underline{a})}\right]=\sum_{\underline{a}\in \underline{A}}p^{\left(\lambda \right)}\left(\underline{a}\right)\varsigma \left(\underline{a}\right),\\ \xi^{\prime \prime }\left(\lambda \right)=-e^{2\xi (\lambda )}\left[\sum_{\underline{a},\underline{b}\in \underline{A}}p\left(\underline{a}\right)p\left(\underline{b}\right)\frac{{\left\{\varsigma \left(\underline{a}\right)-\varsigma \left(\underline{b}\right)\right\}}^{2}}{2}e^{-\lambda \left\{\varsigma \left(\underline{a}\right)+\varsigma \left(\underline{b}\right)\right\}}\right]\\ =-\sum_{\underline{a},\underline{b}\in \underline{A}}p^{\left(\lambda \right)}\left(\underline{a}\right)p^{\left(\lambda \right)}\left(\underline{b}\right)\frac{{\left\{\varsigma \left(\underline{a}\right)-\varsigma \left(\underline{b}\right)\right\}}^{2}}{2}=-\sum_{\underline{a}\in \underline{A}}p^{\left(\lambda \right)}\left(\underline{a}\right)\varsigma^{2}\left(\underline{a}\right)+{\left[\sum_{\underline{a}\in \underline{A}}p^{\left(\lambda \right)}\left(\underline{a}\right)\varsigma \left(\underline{a}\right)\right]}^{2}\leq 0. \end{array}$$

On upper bound of $-\xi^{\prime \prime }\left(\lambda \right)\geq 0$ for λ∈[0,1/2], we have the following chain of inequalities:

(A31) $$\begin{array}{c} -\xi^{\prime \prime }\left(\lambda \right)\overset{\left(a\right)}{\leq }\sum_{\underline{a}\in \underline{A}}p^{\left(\lambda \right)}\left(\underline{a}\right)\varsigma^{2}\left(\underline{a}\right)\overset{\left(b\right)}{=}\sum_{\underline{a}\in \underline{A}}p\left(\underline{a}\right)\varsigma^{2}\left(\underline{a}\right)e^{-\lambda \varsigma \left(\underline{a}\right)+\xi \left(\lambda \right)}\\ =e^{\xi (\lambda )}\sum_{\underline{a}\in \underline{A}}p\left(\underline{a}\right)\sqrt{e^{-2\lambda \varsigma (\underline{a})}}\sqrt{\varsigma^{4}\left(\underline{a}\right)}\overset{\left(c\right)}{\leq }\sqrt{e^{2\xi (\lambda )-\xi (2\lambda )}}\sqrt{\sum_{\underline{a}\in \underline{A}}p\left(\underline{a}\right)\varsigma^{4}\left(\underline{a}\right)}\\ \overset{\left(d\right)}{\leq }\sqrt{e^{2\xi (\lambda )}}\sqrt{\sum_{\underline{a}\in \underline{A}}p\left(\underline{a}\right)\varsigma^{4}\left(\underline{a}\right)}. \end{array}$$

Step (a) follows from Equation (A30). Step (b) follows from Equation (A29). Step (c) follows from Cauchy–Schwarz inequality and Equation (A28). Step (d) follows from that ξ(2λ)≥0 for 2λ∈[0,1]. Note that ξ(λ) exists for λ∈[0,1/2]. Furthermore, we have the following:

$$\sum_{\underline{a}\in \underline{A}}p\left(\underline{a}\right)\varsigma^{4}\left(\underline{a}\right)<\infty .$$

Hence, by Equation (A31), $\xi^{\prime \prime }\left(\lambda \right)$ exists for λ∈[0,1/2]. We next prove Part (d). We derive the lower bound Equation (9) of ${\tilde{\Omega }}^{\left(\mu ,\lambda \right)}\left(p_{XY}\right)$. Fix any (μ,λ)∈[0,1]×[0,1/2] and any $p\in P_{\mathrm{sh}}\left(p_{XY}\right)$. By the Taylor expansion of $\xi \left(\lambda \right)={\tilde{\Omega }}^{\left(\mu ,\lambda \right)}\left(p\right)$ with respect to λ around λ=0, we have that, for any $p\in P_{\mathrm{sh}}\left(p_{XY}\right)$ and for some ν∈[0,λ],

(A32) $$\begin{array}{c} {\tilde{\Omega }}^{\left(\mu ,\lambda \right)}\left(p\right)=\xi \left(0\right)+\xi^{\prime }\left(0\right)\lambda +\frac{1}{2}\xi^{\prime \prime }\left(\nu \right)\lambda^{2}\\ =\lambda E_{p}\left[{\tilde{\omega }}_{p}^{\left(\mu \right)}\left(X,Y,Z|U\right)\right]-\frac{\lambda^{2}}{2}{\mathrm{Var}}_{p^{\left(\nu \right)}}\left[{\tilde{\omega }}_{p}^{\left(\mu \right)}\left(X,Y,Z|U\right)\right]\\ \overset{\left(a\right)}{\geq }\lambda R^{\left(\mu \right)}\left(p_{XY}\right)-\frac{\lambda^{2}}{2}{\mathrm{Var}}_{p^{\left(\nu \right)}}\left[{\tilde{\omega }}_{p}^{\left(\mu \right)}\left(X,Y,Z|U\right)\right]. \end{array}$$

Step (a) follows from $p\in P_{\mathrm{sh}}\left(p_{XY}\right)$,

$$E_{p}\left[{\tilde{\omega }}_{p}^{\left(\mu \right)}\left(X,Y,Z|U\right)\right]=\bar{\mu }I_{p}\left(X;U|Y\right)+\mu E_{p}d\left(X,Z\right),$$

and the definition of $R^{\left(\mu \right)}\left(p_{XY}\right)$. Let $(\nu_{\mathrm{opt}},p_{\mathrm{opt}})\in [0,\lambda ]\times P_{\mathrm{sh}}\left(p_{XY}\right)$ be a pair which attains $\rho^{\left(\mu ,\lambda \right)}\left(p_{XY}\right)$. By this definition, we have that

(A33) $$\begin{array}{c} {\tilde{\Omega }}^{\left(\mu ,\lambda \right)}\left(p_{\mathrm{opt}}\right)={\tilde{\Omega }}^{\left(\mu ,\lambda \right)}\left(p_{XY}\right) \end{array}$$

and that, for any ν∈[0,λ],

(A34) $$\begin{array}{c} {\mathrm{Var}}_{p_{\mathrm{opt}}^{\left(\nu \right)}}\left[\omega_{p_{\mathrm{opt}}}^{\left(\mu \right)}\left(X,Y,Z|U\right)\right]\leq {\mathrm{Var}}_{p_{\mathrm{opt}}^{\left(\nu_{\mathrm{opt}}\right)}}\left[\omega_{p_{\mathrm{opt}}}^{\left(\mu \right)}\left(X,Y,Z|U\right)\right]=\rho^{\left(\mu ,\lambda \right)}\left(p_{XY}\right). \end{array}$$

On lower bounds of $\Omega^{\left(\mu ,\lambda \right)}\left(p_{XY}\right)$, we have the following chain of inequalities:

$$\begin{array}{cc} & {\tilde{\Omega }}^{\left(\mu ,\lambda \right)}\left(p_{XY}\right)\overset{\left(a\right)}{=}{\tilde{\Omega }}^{\left(\mu ,\lambda \right)}\left(p_{\mathrm{opt}}\right)\overset{\left(b\right)}{\geq }\lambda R^{\left(\mu \right)}\left(p_{XY}\right)-\frac{\lambda^{2}}{2}{\mathrm{Var}}_{p_{\mathrm{opt}}^{\left(\nu \right)}}\left[{\tilde{\omega }}_{p_{\mathrm{opt}}}^{\left(\mu \right)}\left(X,Y,Z|U\right)\right]\\ \overset{\left(c\right)}{\geq } & \lambda R^{\left(\mu \right)}\left(p_{XY}\right)-\frac{\lambda^{2}}{2}\rho^{\left(\mu ,\lambda \right)}\left(p_{XY}\right)\overset{\left(d\right)}{\geq }\lambda R^{\left(\mu \right)}\left(p_{XY}\right)-\frac{\lambda^{2}}{2}\rho \left(p_{XY}\right). \end{array}$$

Step (a) follows from Equation (A33). Step (b) follows from Equation (A32). Step (c) follows from Equation (A34). Step (d) follows from the definition of $\rho (p_{XY})$. ☐

We next prove Part (e). For the proof we use the following lemma.

**Lemma** **A4.**
*When τ∈(0,(1/2)ρ], the maximum of*

$$\frac{1}{5+2\lambda }\left\{-\frac{1}{2}\rho \lambda^{2}+\tau \lambda \right\}$$

*for λ∈(0,1/2] is attained by the positive $\lambda_{0}$ satisfying*

(A35) $$\vartheta \left(\lambda_{0}\right):=\frac{1}{5}\lambda_{0}^{2}+\lambda_{0}=\frac{\tau }{\rho }.$$

*Let g(a) be the inverse function of ϑ(a) for a≥0. Then, the condition of Equation (A35) is equivalent to $\lambda_{0}=g\left(\frac{\tau }{\rho }\right)$. The maximum is given by*

$$\frac{1}{5+2\lambda_{0}}\left\{-\frac{1}{2}\rho \lambda_{0}^{2}+\tau \lambda_{0}\right\}=\frac{\rho }{10}\lambda_{0}^{2}=\frac{\rho }{10}g^{2}\left(\frac{\tau }{\rho }\right).$$

By an elementary computation we can prove this lemma. We omit the detail.

**Proof** **of** **Property** **4** **Part** **(e):** By the hyperplane expression $R_{\mathrm{sh}}\left(p_{XY}\right)$ of $R(p_{XY})$ stated Property 3 we have that when $\left(R+\tau ,\Delta +\tau \right)\notin R\left(p_{XY}\right)$, we have

$${\bar{\mu }}^{*}\left(R+\tau \right)+\mu^{*}\left(\Delta +\tau \right)<R^{\left(\mu^{*}\right)}\left(p_{XY}\right)$$

or equivalent to

(A36) $$R^{\left(\mu^{*}\right)}\left(p_{XY}\right)-\left({\bar{\mu }}^{*}R+\mu^{*}\Delta \right)>\tau$$

for some $\mu^{*}\in \left[0,1\right]$. Then, for each positive τ, we have the following chain of inequalities:

$$\begin{array}{c} \tilde{F}\left(R,\Delta |p_{XY}\right)\geq \underset{\lambda \in (0,1/2]}{\sup}{\tilde{F}}^{\left(\mu^{*},\lambda \right)}\left({\bar{\mu }}^{*}R+\mu^{*}\Delta |p_{XY}\right)\\ =\underset{\lambda \in (0,1/2]}{\sup}\frac{{\tilde{\Omega }}^{\left(\mu^{*},\lambda \right)}\left(p_{XY}\right)-\lambda \left({\bar{\mu }}^{*}R+\mu^{*}\Delta \right)}{5+\lambda (1+{\bar{\mu }}^{*})}\\ \overset{\left(a\right)}{\geq }\underset{\lambda \in (0,1/2]}{\sup}\frac{1}{5+2\lambda }\left\{-\frac{1}{2}\rho \lambda^{2}\right.+\lambda R^{\left(\mu^{*}\right)}\left(p_{XY}\right)-\lambda \left({\bar{\mu }}^{*}R+\mu^{*}\Delta \right)\}\\ \overset{\left(b\right)}{>}\underset{\lambda \in (0,1/2]}{\sup}\frac{1}{5+2\lambda }\left\{-\frac{1}{2}\rho \lambda^{2}+\tau \lambda \right\}\overset{\left(c\right)}{=}\frac{\rho }{10}g^{2}\left(\frac{\tau }{\rho }\right). \end{array}$$

Step (a) follows from Property 4 Part (d). Step (b) follows from Equation (A36). Step (c) follows from Lemma A4. ☐

## Appendix G. Proof of Lemma 1

To prove Lemma 1, we prepare a lemma. Set

$$\begin{array}{cc} {\tilde{A}}_{n} & :=\left\{x^{n}:\frac{1}{n}\log\frac{p_{X^{n}}\left(x^{n}\right)}{Q_{X^{n}}^{\left(i\right)}\left(x^{n}\right)}\geq -\eta \right\},\\ A_{n} & :={\tilde{A}}_{n}\times M_{n}\times Y^{n}\times Z^{n},\;A_{n}^{c}:={\tilde{A}}_{n}^{c}\times M_{n}\times Y^{n}\times Z^{n},\\ {\tilde{B}}_{n} & :=\left\{\left(s,x^{n},y^{n}\right):\frac{1}{n}\log\frac{p_{Y^{n}|X^{n}}\left(y^{n}|x^{n}\right)}{Q_{Y^{n}|X^{n}S_{n}}^{\left(\mathrm{ii}\right)}\left(y^{n}|x^{n},s\right)}\geq -\eta \right\},\\ B_{n} & :={\tilde{B}}_{n}\times Z^{n},B_{n}^{c}:={\tilde{B}}_{n}^{c}\times Z^{n},\\ C_{n} & :=\left\{\left(s,x^{n},y^{n},z^{n}\right):\frac{1}{n}\log\frac{p_{X^{n}|S_{n}Y^{n}}\left(x^{n}|s,y^{n}\right)}{Q_{X^{n}|S_{n}Y^{n}Z^{n}}^{\left(\mathrm{iii}\right)}\left(x^{n}|s,y^{n},z^{n}\right)}\geq -\eta \right\},\\ {\tilde{D}}_{n} & :=\{\left(s,x^{n},y^{n}\right):s=\varphi^{\left(n\right)}\left(x^{n}\right),Q_{X^{n}|S_{n}Y^{n}}^{\left(\mathrm{iv}\right)}\left(x^{n}|s,y^{n}\right)\leq M_{n}e^{n\eta }p_{X^{n}|Y^{n}}\left(x^{n}|y^{n}\right)\},\\ D_{n} & :={\tilde{D}}_{n}\times Z^{n},D_{n}^{c}:={\tilde{D}}_{n}^{c}\times Z^{n}. \end{array}$$

Then, we have the following lemma:

**Lemma** **A5.**

$$\begin{array}{c} p_{S_{n}X^{n}Y^{n}Z^{n}}\left(A_{n}^{c}\right)\leq e^{-n\eta },p_{S_{n}X^{n}Y^{n}Z^{n}}\left(B_{n}^{c}\right)\leq e^{-n\eta },\\ p_{S_{n}X^{n}Y^{n}Z^{n}}\left(C_{n}^{c}\right)\leq e^{-n\eta },p_{S_{n}X^{n}Y^{n}Z^{n}}\left(D_{n}^{c}\right)\leq e^{-n\eta }. \end{array}$$

**Proof.** We first prove the first inequality. We have the following chain of inequalities:

$$\begin{array}{cc} & p_{S_{n}X^{n}Y^{n}Z^{n}}\left(A_{n}^{c}\right)=p_{X^{n}}\left({\tilde{A}}_{n}^{c}\right)=\sum_{x^{n}\in {\tilde{A}}_{n}^{c}}p_{X_{n}}\left(x^{n}\right)\\ \overset{\left(a\right)}{\leq } & \sum_{x^{n}\in {\tilde{A}}_{n}^{c}}e^{-n\eta }Q_{X^{n}}^{\left(i\right)}\left(x^{n}\right)\leq e^{-n\eta }\sum_{x^{n}}Q_{X^{n}}^{\left(i\right)}\left(x^{n}\right)=e^{-n\eta }. \end{array}$$

Step (a) follows from the definition of $A_{n}$. We next prove the second inequality. We have the following chain of inequalities:

$$\begin{array}{c} p_{S_{n}X^{n}Y^{n}Z^{n}}\left(B_{n}^{c}\right)=p_{S_{n}X^{n}Y^{n}}\left({\tilde{B}}_{n}^{c}\right)\overset{\left(a\right)}{=}\sum_{\left(s,x^{n},y^{n}\right)\in {\tilde{B}}_{n}^{c}}p_{S_{n}X^{n}}\left(s,x^{n}\right)p_{Y^{n}|X^{n}}\left(y^{n}|x^{n}\right)\\ \overset{\left(b\right)}{\leq }\sum_{\left(s,x^{n},y^{n}\right)\in {\tilde{B}}_{n}^{c}}e^{-n\eta }p_{S_{n}X^{n}}\left(s,x^{n}\right)Q_{Y^{n}|S_{n}X^{n}}^{\left(\mathrm{ii}\right)}\left(y^{n}|s,x^{n}\right)\\ \leq e^{-n\eta }\sum_{s,x^{n},y^{n}}p_{S_{n}X^{n}}\left(s,x^{n}\right)Q_{Y^{n}|S_{n}X^{n}}^{\left(\mathrm{ii}\right)}\left(y^{n}|s,x^{n}\right)=e^{-n\eta }. \end{array}$$

Step (a) follows from the Markov chain $S_{n}↔X^{n}↔Y^{n}$. Step (b) follows from the definition of $B_{n}$. On the third inequality, we have the following chain of inequalities:

$$\begin{array}{c} p_{S_{n}X^{n}Y^{n}Z^{n}}\left(C_{n}^{c}\right)\overset{\left(a\right)}{=}\sum_{\left(s,x^{n},y^{n},z^{n}\right)\in C_{n}^{c}}p_{X^{n}|S_{n}Y^{n}}\left(x^{n}|s,y^{n}\right)p_{S_{n}Y^{n}Z^{n}}\left(s,y^{n},z^{n}\right)\\ \overset{\left(b\right)}{\leq }\sum_{\left(s,x^{n},y^{n},z^{n}\right)\in C_{n}^{c}}e^{-n\eta }Q_{X^{n}|S_{n}Y^{n}Z^{n}}^{\left(\mathrm{iii}\right)}\left(x^{n}|s,y^{n},z^{n}\right)p_{S_{n}Y^{n}Z^{n}}\left(s,y^{n},z^{n}\right)\\ \leq e^{-n\eta }\sum_{s,x^{n},y^{n},z^{n}}Q_{X^{n}|S_{n}Y^{n}Z^{n}}^{\left(\mathrm{iii}\right)}\left(x^{n}|s,y^{n},z^{n}\right)p_{S_{n}Y^{n}Z^{n}}\left(s,y^{n},z^{n}\right)=e^{-n\eta }. \end{array}$$

Step (a) follows from the Markov chain $X^{n}↔S_{n}Y^{n}↔Z^{n}$. Step (b) follows from the definition of $C_{n}$. We finally prove the fourth inequality. We have the following chain of inequalities:

pSnXnYnZn(Dnc)=pSnXnYn(D˜nc)=∑s∈Mn∑(xn,yn):φ(n)(xn)=s,pXn|Yn(xn|yn)≤(1/Mn)e−nη×QXn|Sn,Yn(iv)(xn|s,yn)pXn|Yn(xn|yn)pYn(yn)≤e−nηMn∑s∈Mn∑(xn,yn):φ(n)(xn)=s,pXn|Yn(xn|yn)≤(1/Mn)e−nη×QXn|SnYn(iv)(xn|s,yn)QXn|SnYn(iv)(xn|s,yn)pYn(yn)≤e−nηMn∑s∈Mn∑xn,ynQXn|SnYn(iv)(xn|s,yn)pYn(yn)=e−nη.

☐

**Proof** **of** **Lemma** **1:** We set

$$E_{n}:=\left\{\left(s,x^{n},y^{n},z^{n}\right):\frac{1}{n}d\left(X^{n},Z^{n}\right)\leq \Delta \right\}.$$

Set $R^{\left(n\right)}:=\left(1/n\right)\log M_{n}$. By definition, we have

(A37) $$\begin{array}{c} p_{S_{n}X^{n}Y^{n}Z^{n}}\left(A_{n}\cap B_{n}\cap C_{n}\cap D_{n}\cap E_{n}\right)\\ \begin{array}{ccc} =p_{S_{n}X^{n}Y^{n}Z^{n}}\{\eta & \geq & \frac{1}{n}\log\frac{Q_{X^{n}}^{\left(i\right)}\left(X^{n}\right)}{p_{X^{n}}\left(X^{n}\right)},\\ \eta & \geq & \frac{1}{n}\log\frac{Q_{Y^{n}|X^{n}S}^{\left(\mathrm{ii}\right)}\left(Y^{n}|X^{n}S\right)}{p_{Y^{n}|X^{n}}\left(Y^{n}|X^{n}\right)},\\ \eta & \geq & \frac{1}{n}\log\frac{Q_{X^{n}|S_{n}Y^{n}Z^{n}}^{\left(\mathrm{iii}\right)}\left(X^{n}|S_{n}Y^{n}Z^{n}\right)}{p_{X^{n}|S_{n}Y^{n}}\left(X^{n}|S_{n}Y^{n}\right)},\\ R^{\left(n\right)}+\eta & \geq & \frac{1}{n}\log\frac{Q_{X^{n}|S_{n}Y^{n}}^{\left(\mathrm{iv}\right)}\left(X^{n}|S_{n}Y^{n}\right)}{p_{X^{n}|Y^{n}}\left(X^{n}|Y^{n}\right)},\\ \Delta & \geq & \left.\frac{1}{n}\log\exp\left\{d(X^{n},Z^{n})\right\}\right\}. \end{array} \end{array}$$

Then, for any $(\varphi^{\left(n\right)}$, $\psi^{\left(n\right)})$ satisfying

$$R^{\left(n\right)}=\frac{1}{n}\log M_{n}\leq R,$$

we have

(A38) $$\begin{array}{c} p_{S_{n}X^{n}Y^{n}Z^{n}}\left(A_{n}\cap B_{n}\cap C_{n}\cap D_{n}\cap E_{n}\right)\\ \begin{array}{ccc} \leq p_{S_{n}X^{n}Y^{n}Z^{n}}\{\eta & \geq & \frac{1}{n}\log\frac{Q_{X^{n}}^{\left(i\right)}\left(X^{n}\right)}{p_{X^{n}}\left(X^{n}\right)},\\ \eta & \geq & \frac{1}{n}\log\frac{Q_{Y^{n}|X^{n}S}^{\left(\mathrm{ii}\right)}\left(Y^{n}|X^{n}S\right)}{p_{Y^{n}|X^{n}}\left(Y^{n}|X^{n}\right)},\\ \eta & \geq & \frac{1}{n}\log\frac{Q_{X^{n}|S_{n}Y^{n}Z^{n}}^{\left(\mathrm{iii}\right)}\left(X^{n}|S_{n}Y^{n}Z^{n}\right)}{p_{X^{n}|S_{n}Y^{n}}\left(X^{n}|S_{n}Y^{n}\right)},\\ R+\eta & \geq & \frac{1}{n}\log\frac{Q_{X^{n}|S_{n}Y^{n}}^{\left(\mathrm{iv}\right)}\left(X^{n}|S_{n}Y^{n}\right)}{p_{X^{n}|Y^{n}}\left(X^{n}|Y^{n}\right)},\\ \Delta & \geq & \left.\frac{1}{n}\log\exp\left\{d(X^{n},Z^{n})\right\}\right\}. \end{array} \end{array}$$

Hence, it suffices to show

$$\begin{array}{ccc} P_{c}^{\left(n\right)}\left(\varphi_{1}^{\left(n\right)},\varphi_{2}^{\left(n\right)},\psi^{\left(n\right)};\Delta \right) & \leq & p_{S_{n}X^{n}Y^{n}}\left(A_{n}\cap B_{n}\cap C_{n}\cap D_{n}\cap E_{n}\right)+4e^{-n\eta } \end{array}$$

to prove Lemma 1. By definition, we have

$$\begin{array}{c} P_{c}^{\left(n\right)}\left(\varphi^{\left(n\right)},\psi^{\left(n\right)};\Delta \right)=p_{S_{n}X^{n}Y^{n}Z^{n}}\left(E_{n}\right). \end{array}$$

Then, we have the following:

$$\begin{array}{cc} & P_{c}^{\left(n\right)}\left(\varphi^{\left(n\right)},\psi^{\left(n\right)};\Delta \right)=p_{S_{n}X^{n}Y^{n}Z^{n}}\left(E_{n}\right)\\ = & p_{S_{n}X^{n}Y^{n}Z^{n}}\left(A_{n}\cap B_{n}\cap C_{n}\cap D_{n}\cap E_{n}\right)+p_{S_{n}X^{n}Y^{n}Z^{n}}\left({\left[A_{n}\cap B_{n}\cap C_{n}\cap D_{n}\right]}^{c}\cap E_{n}\right)\\ \leq & p_{S_{n}X^{n}Y^{n}Z^{n}}\left(A_{n}\cap B_{n}\cap C_{n}\cap D_{n}\cap E_{n}\right)+p_{S_{n}X^{n}Y^{n}Z^{n}}\left(A_{n}^{c}\right)+p_{S_{n}X^{n}Y^{n}Z^{n}}\left(B_{n}^{c}\right)\\ & +p_{S_{n}X^{n}Y^{n}Z^{n}}\left(C_{n}^{c}\right)+p_{S_{n}X^{n}Y^{n}Z^{n}}\left(D_{n}^{c}\right)\\ \overset{\left(a\right)}{\leq } & p_{S_{n}X^{n}Y^{n}Z^{n}}\left(A_{n}\cap B_{n}\cap C_{n}\cap D_{n}\cap E_{n}\right)+4e^{-n\eta }. \end{array}$$

Step (a) follows from Lemma A5. ☐

## Appendix H. Proof of Lemma 2

In this Appendix, we prove Lemma 2.

**Proof** **of** **Lemma** **2:** We have the following chain of inequalities:

$$\begin{array}{cc} & I\left(X_{t};Y^{t-1}|S_{n}X_{t-1}Y_{t}^{n}\right)=H\left(Y^{t-1}|S_{n}X^{t-1}Y_{t}^{n}\right)-H\left(Y^{t-1}|S_{n}X^{t}Y_{t}^{n}\right)\\ \leq & H\left(Y^{t-1}|X^{t-1}\right)-H\left(Y^{t-1}|S_{n}X^{n}Y_{t}^{n}\right)\overset{\left(a\right)}{=}H\left(Y^{t-1}|X^{t-1}\right)-H\left(Y^{t-1}|X^{n}Y_{t}^{n}\right)\\ \overset{\left(b\right)}{=} & H\left(Y^{t-1}|X^{t-1}\right)-H\left(Y^{t-1}|X^{t-1}\right)=0. \end{array}$$

Step (a) follows from that $S_{n}=\varphi^{\left(n\right)}\left(X^{n}\right)$ is a function of $X^{n}$. Step (b) follows from the memoryless property of the information source ${\left\{\left(X_{t},Y_{t}\right)\right\}}_{t=1}^{\infty }$. ☐

## Appendix I. Proof of Lemma 5

In this Appendix, we prove Equations (32) and (33) in Lemma 5.

**Proofs** **Equations** **(32)** **and** **(33)** **in** **Lemma** **5:** By the definition of $p_{X^{t}Z^{t}|S_{n}Y^{n},q^{t}}^{\left(\mu ,\lambda ,\theta \right)}$$\left(x^{t},z^{t}|s,y^{n}\right)$, for t=1,2,⋯,n, we have

(A39) $$\begin{array}{ccc} p_{X^{t}Z^{t}|S_{n}Y^{n};F^{t}}^{\left(\mu ,\lambda ,\theta \right)}\left(x^{t},z^{t}|s,y^{n}\right) & = & C_{t}^{-1}\left(s,y^{n}\right)p_{X^{t}Z^{t}|S_{n}Y^{n}}\left(x^{t},z^{t}|s,y^{n}\right)\prod_{i=1}^{t}f_{F_{i}}^{\left(\mu ,\lambda ,\theta \right)}\left(x_{i},y_{i},z_{i}|u_{i}\right). \end{array}$$

Then, we have the following chain of equalities:

(A40) $$\begin{array}{c} p_{X^{t}Z^{t}|S_{n}Y^{n};F^{t}}^{\left(\mu ,\lambda ,\theta \right)}\left(x^{t},z^{t}|s,y^{n}\right)\overset{\left(a\right)}{=}C_{t}^{-1}\left(s,y^{n}\right)p_{X^{t}Z^{t}|S_{n}Y^{n}}\left(x^{t},z^{t}|s,y^{n}\right)\prod_{i=1}^{t}f_{F_{i}}^{\left(\mu ,\lambda ,\theta \right)}\left(x_{i},y_{i},z_{i}|u_{i}\right)\\ =C_{t}^{-1}\left(s,y^{n}\right)p_{X^{t-1}Z^{t-1}|S_{n}Y^{n}}\left(x^{t-1},z^{t-1}|s,y^{n}\right)\prod_{i=1}^{t-1}f_{F_{i}}^{\left(\mu ,\lambda ,\theta \right)}\left(x_{i},y_{i},z_{i}|u_{i}\right)\\ \;\times p_{X_{t}\left|Z_{t}\right|X^{t-1}Z^{t-1}SY^{n}}\left(x_{t},z_{t}|x^{t-1},z^{t-1},s,y^{n}\right)f_{F_{t}}^{\left(\mu ,\lambda ,\theta \right)}\left(x_{t},y_{t}|u_{t}\right)\\ \overset{\left(b\right)}{=}\frac{C_{t-1}\left(s,y^{n}\right)}{C_{t}\left(s,y^{n}\right)}p_{X^{t-1}Z^{t-1}|S_{n}Y^{n};F^{t-1}}^{\left(\mu ,\lambda ,\theta \right)}\left(x^{t-1},z^{t-1}|s,y^{n}\right)\\ \;\times p_{X_{t}\left|Z_{t}\right|X^{t-1}Z^{t-1}S_{n}Y^{n}}\left(x_{t},z_{t}|x^{t-1},z^{t-1},s,y^{n}\right)f_{F_{t}}^{\left(\mu ,\lambda ,\theta \right)}\left(x_{t},y_{t},z_{t}|u_{t}\right)\\ ={\left(\Phi_{t}^{\left(\mu ,\lambda ,\theta \right)}\left(s,y^{n}\right)\right)}^{-1}p_{X^{t-1}Z^{t-1}|S_{n}Y^{n};F^{t-1}}^{\left(\mu ,\lambda ,\theta \right)}\left(x_{t},y_{t},z_{t}|u_{t}\right)\\ \;\times p_{X_{t}\left|Z_{t}\right|X^{t-1}Z^{t-1}S_{n}Y^{n}}\left(x_{t},z_{t}|x^{t-1},z^{t-1},s,y^{n}\right)f_{F_{t}}^{\left(\mu ,\lambda ,\theta \right)}\left(x_{t},y_{t},z_{t}|u_{t}\right). \end{array}$$

Steps (a) and (b) follow from Equation (A39). From Equation (A40), we have

(A41) $$\begin{array}{c} \Phi_{t,q^{t}}^{\left(\mu ,\lambda ,\theta \right)}\left(s,y^{n}\right)p_{X^{t}Z^{t}|S_{n}Y^{n}}^{\left(\mu ,\lambda ,\theta \right)}\left(x^{t},z^{t}|s,y^{n}\right)\\ =p_{X^{t-1}Z^{t-1}|S_{n}Y^{n};F^{t-1}}^{\left(\mu ,\lambda ,\theta \right)}\left(x^{t-1},z^{t-1}|s,y^{n}\right) \end{array}$$

(A42) $$\begin{array}{c} \;\times p_{X_{t}Z_{t}|X^{t-1}Z^{t-1}S_{n}Y^{n}}\left(x_{t},z_{t}|x^{t-1},z^{t-1},s,y^{n}\right)f_{F_{t}}^{\left(\mu ,\lambda ,\theta \right)}\left(x_{t},y_{t},z_{t}|u_{t}\right). \end{array}$$

Taking summations of Equation (A41) and (A42) with respect to $x^{t},z^{t}$, we obtain

$$\begin{array}{c} \Phi_{t,F^{t}}^{\left(\mu ,\lambda ,\theta \right)}\left(s,y^{n}\right)=\sum_{x^{t},z^{t}}p_{X^{t-1}Z^{t-1}|S_{n}Y^{n};F^{t-1}}^{\left(\mu ,\lambda ,\theta \right)}\left(x^{t-1},z^{t-1}|s,y^{n}\right)\\ \;\times p_{X_{t}Z_{t}|X^{t-1}Z^{t-1}S_{n}Y^{n}}\left(x_{t},z_{t}|x^{t-1},z^{t-1},s,y^{n}\right)f_{F_{t}}^{\left(\mu ,\lambda ,\theta \right)}\left(x_{t},y_{t},z_{t}|u_{t}\right), \end{array}$$

completing the proof. ☐
